# Intersectoral collaboration for the prevention and control of vector borne diseases to support the implementation of a global strategy: A systematic review

**DOI:** 10.1371/journal.pone.0204659

**Published:** 2018-10-10

**Authors:** Herdiana Herdiana, Jana Fitria Kartika Sari, Maxine Whittaker

**Affiliations:** 1 Paritrana Asia Foundation, Jakarta, Indonesia; 2 College of Public Health, Medical and Veterinary Sciences, The Australian Institute of Tropical Health and Medicine (AITHM) at James Cook University, Jakarta, Australia; National Institute for Communicable Diseases, SOUTH AFRICA

## Abstract

**Introduction:**

Vector Borne Diseases (VBDs) have a major impact on public health and socio-economic development. Inter-sectoral collaboration was recommended as one of the key elements of Integrated Vector Management (IVM), however limited evidence measures the effect and contribution of intersectoral approaches including but not only IVM. This systematic review aims to assess the existing evidence on all forms of inter-sectoral collaboration in VBD control and prevention, identify any gaps and develop a framework from a global perspective.

**Methods:**

Articles were identified through a search of PUBMED, Science of Direct, Web of Knowledge, Google Scholar and WHO archives using key words and excluded duplications (n = 2,034). The exclusion of non-VBDs control and prevention interventions resulted in 194 eligible titles/abstract/keywords for full text assessment. Further exclusion of non-peer reviewed articles, non-declaration of ethical clearance, reviews and expert opinion articles resulted in 50 articles finally being included for analysis with the extraction of data on outcome, factor/s influencing the effectiveness, indicators of collaboration and sustainability.

**Results:**

Of the 50 articles included in the analysis, 19 articles were categorized as of moderate-strong quality. All articles compared pre- and post-intervention outcomes against disease or vector variables. Three papers included outcome variables on intersectoral collaboration and participation indicator. However, no paper undertook component analysis by different sectors or different activities. Only one paper compared cost data for community-intersectoral intervention for IRS and traditional “vertical” IRS. Six factors were identified as influencing the effectiveness of inter-sectoral collaboration. Five of six factors are the main ones, namely the approach (37/47), resources (34/47), relationships (33/47), management (29/47) and shared vision (20/47) factors. A conceptual framework has been developed based on this review.

**Conclusion:**

This review shows the importance of inter-sectoral collaboration to reduce VBDs or vector densities. However, very few studies measured how much inter-sectoral collaboration contributes to the impact. Further high-quality studies using inter-sectoral collaboration indicators are recommended to be undertaken.

## Introduction

### Burden of VBDs worldwide

Vector-borne diseases (VBDs) contribute significantly to the global burden of diseases and have a major impact on public health and socio-economic development. In 2015, VBDs accounted for approximately 17% of the estimated communicable diseases cases worldwide [1]. VBDs are defined as illnesses caused by viruses, bacteria and parasites in human populations carried by a vector. The vectors incriminated in carrying these infectious agents are mosquitoes, ticks, sandflies, fleas, triatomine bugs, blackflies, tsetse flies, mites, lice and some freshwater aquatic snails. Every year there are more than 1 billion cases and over 1 million deaths from vector-borne diseases such as malaria, dengue, Zika, schistosomiasis, human African trypanosomiasis, leishmaniasis, Chagas disease, yellow fever, Japanese encephalitis and onchocerciasis, globally. The distribution of these diseases is mostly in tropical and sub-tropical countries and determined by a complex dynamic of environmental and social factors [2].

The burden of VBDs is highest in tropical and subtropical areas and usually affects the poorest populations. Currently, the increase in global population movement, unplanned urbanization, climate change and other environmental and social challenges determine the distribution of these diseases. Changes in agricultural practices due to factors such as variation in temperature and rainfall, forest clearing, growth of urban areas including slums, lacking reliable piped water or adequate solid waste management become factors that influence the range of vector populations and the transmission patterns of vector-borne diseases [3].

To control the VBDs, many countries continue to face technical and operational challenges [4–7]. In 2004, the World Health Organization (WHO) adopted the integrated vector management (IVM) global for all vector-borne diseases control program [8]. One key element of IVM is collaboration between an intra-health sector and non-health (inter-) sectors. IVM is defined as a “rational decision-making process for the optimal use of resources for vector control” [8]. IVM aims to plan, deliver, monitor and evaluate cost-effective intervention, as well as to use combinations of vector control measures. A measurable impact on the risks of transmission, inter-sectorality and partnership are also important factors of IVM [9]. Furthermore, according to the recent global mapping of VBDs distribution, many countries suffer from more than one vector borne disease. Thus, integrated vector control interventions can be an effective and efficient solution to control several diseases at the same time alongside with the strengthening intersectoral collaboration [10]. Prevention of vector borne diseases can also include treatment—not just management of vectors, and the experience of intersectoral approaches to this are also limited in assessment.

### VBDs approaches

The importance of intersectoral collaboration has been acknowledged for a long time. Roles in VBDs control outside the health sector had been initiated since the sixth century when agricultural fields were drained resulting in a positive impact upon the health and wealth of population [11] (even if the mechanism for this outcome was not understood scientifically) and later examples like the draining of the Pontine marches in Italy [12]. The role of involvement of engineers in the design of mosquito control interventions in many countries was activated after the relationship between mosquito and human health had been discovered in 1897. The engineering involvement included drainage, land filling, constructed canal, irrigation networks, removal vegetation, and managed stream water flow [13].

In some countries, intersectoral collaboration including public-private partnerships has proven itself to be a key intervention in reaching zero cases of VBDs [4, 14–16]. At the ‘end game‘ of the VBDs control efforts, inter-sectoral collaboration becomes crucial for scaling up and ensuring sustainability of control and often prevention of re-introduction of disease efforts. Although inter-sectoral collaboration is recommended to reduce the burden of VBDs, the type of collaboration and segregation of duties, responsibilities, and resources seem unclear [16]. Many VBDs control programmes are organized under the Ministries of Health (MoH) [17], although exceptions, like the Singaporean *Aedes* program through the National Environmental agency exist [18], and are mostly vertical programs. Limited, or even no, institutional collaboration with other government bodies (e.g. environment, education, agriculture and tourism), municipal entities (e.g. engineering, sanitation and water resources) or links to stakeholders and community leaders (e.g. businesses, educators, community groups and NGO's) are evident [16].

Some factors affecting the success of intersectoral collaboration include strengthened channels of communication among policymakers, VBDs control programme managers and other IVM partners; effective dialogue between the health, settlement planning sectors, environmental agencies (with concerns such as wetland conservation), academic institutions (for providing research support) and local communities [19]. Intersectoral collaboration may also support case detection and diagnosis and treatment, vector control and surveillance/response activities, harnessing of political support, partnerships in research and development, and the development of innovative financing mechanisms and health systems initiatives [16].

### Defining intersectoral collaboration

The definition of intersectoral collaboration (ISC) has evolved over the time, though some common elements can be identified. The concept of ISC was highlighted by WHO in the Declaration of Alma Ata 1978 as involving all related sectors and aspects of national and community development to undertake collective actions performing different roles, especially for Primary Health Centres (PHCs) [20]. In 1997, the concept of inter-sectoral action for health (IAH) promoted by WHO, was defined as “*a recognised relationship between health sector and another sector to take action on an issue to achieve health outcome to be more effective*, *efficient or sustainable*”[21]. The IAH concept further emphasized that the collaboration should be a managed process not only a conceptual one. The 1998 WHO Health Promotion Glossary defined ISC as “*cooperation between different sectors of society such as the public sector*, *civil society and the private sector*” [22].

The interest to collaborate more inter-and intra-sectorally has increased since the Ottawa Charter for improving population health, and supported by the recent understanding of how determinants of health such as social, biology, environment, and economic factors as well as traditional determinants of health such as genetic and personal health services act together [21]. The reason there is interest in the health sector working together with other sectors can be summarized as realizing that health is not only the responsibility of health sector; and a need to address the persistence of health inequalities and equity issues. More evidence is required on the enabling factors for effective intersectoral collaboration; and what are the positive impacts of intersectoral action [21].

Historically, ISC was initiated for primary health care and health promotion. However, there are broader examples from health programs that have successful utilized ISC to benefit not only the health sector but also other sectors such as education with school health programs, and city planning with healthy city programs. The conference of intersectoral action for health (IAH) identified the key factors required to achieve common vision, values and goals with the availability of key resources such as human, technical and financial. The key factors of successful IAH are “1). Leadership, champion, catalyst; 2) Analysis or priority-setting; 3). Mutually beneficial relationships; 4). Integrated action either in macro and micro level; 5). Variation of institutional long-term policy; 6) Institutionalizing of health impact or gain assessment; 7). Training, tools, and capacity development; 8). Coordinating and integrating mechanism, partnering; and 9). Social mobilization or community empowerment” [21]. It is interesting to note that ISC was an important founding principles for the development of the Roll Back Malaria Partnership [23].

A conceptual scheme of different forms of integration was suggested by Axelsson R. and Axelsson S.B. in 2006 [24]. It assessed integration based upon degrees of vertical and horizontal integration, across the variables of coordination (high degree of vertical integration with low degree of horizontal integration), cooperation (high degree both vertical and horizontal integration), contracting (low degree both vertical and horizontal integration), and collaboration (high degree horizontal integration with low degree of vertical integration). They elaborated upon collaboration by adding variables as to i) if the integration occurred through voluntary agreement; ii) if mutual adjustments between the sectors or organizations involved was undertaken, iii) evidence of a willingness to work together and iv) the levels of required intensive contacts and communications among the member of organizations [24]. Though many terms have been used around this theme such as collaboration, partnership, cooperation, consolidation, this review used all the terms in the search strategy in order to obtain as much information as possible to understanding the ISC on VBDs control and prevention.

### Intersectoral collaboration in VBDs

The epidemiology of VBDs involves causative agents (parasite, bacterial, virus etc), hosts (primary, intermediate, dead-end, accidental), vectors (mosquitoes, ticks, lice, flies, sand-flies, fleas, triatomine bugs and some freshwater aquatic snails) and environmental factors [1, 2]. Moreover, with a growing understanding the social and economic determinants of VBDs, the overlapping distribution of VBDs across the countries, and the complexity of and inter-relationships between drivers of VBDs, means that the control and prevention of these diseases cannot be tackled by the health sector alone—many factors lie within other sectors’ responsibilities [1, 2]. The recently approved Global Vector Control Response 2017–2030 document [1] by the 70th session of the World Health Assembly noted that ISC is one of the important pillars of action to reduce the human burden caused by VBDs. This agenda of multi-sectoral collaboration for VBDs control and prevention is also being promoted by WHO and partners [1]. Using malaria as an example to build the multi-sectoral collaboration framework [25, 26], can provide guidance for other VBDs control and prevention interventions.

Therefore, this systematic review aims to assess the existing evidence on inter-sectoral collaboration in VBDs control and prevention, identify any gaps in the evidence base and develop a framework for inter-sector collaboration in VBDs from a global perspective.

## Methods

### Search strategy

Overall this review process followed five steps:

1. Framing questions for a review;
2. Identifying relevant work;
3. Assessing the quality of studies;
4. Summarizing the evidence; and
5. Interpreting the findings [27].

This study was limited to inter-sectoral collaboration between health sectors and other sectors such as inter-government or inter-ministerial agencies, private sectors, NGOs and CSOs, regional and international partners.

One reviewer searched peer-review published data sources including quantitative and qualitative research, case studies, and case reports. The literature search used PubMed, ScienceDirect, Google scholar and WHO archives from period 1 January 1970 to 30 June 2017 and in English full text. The search terms used in this review were: (public-private OR inter-sectoral OR multi-sectoral) AND (partnerships OR collaboration OR consolidation OR cooperation OR planning) AND (“vector borne diseases” OR “vector control” OR ‘vector management” OR “disease control” OR malaria OR dengue OR Schistosomiasis OR Leishmaniasis OR “Chaga disease” OR”lymphatic filariasis” OR zika OR “yellow fever”).

### Study selection

**Fig 1:**
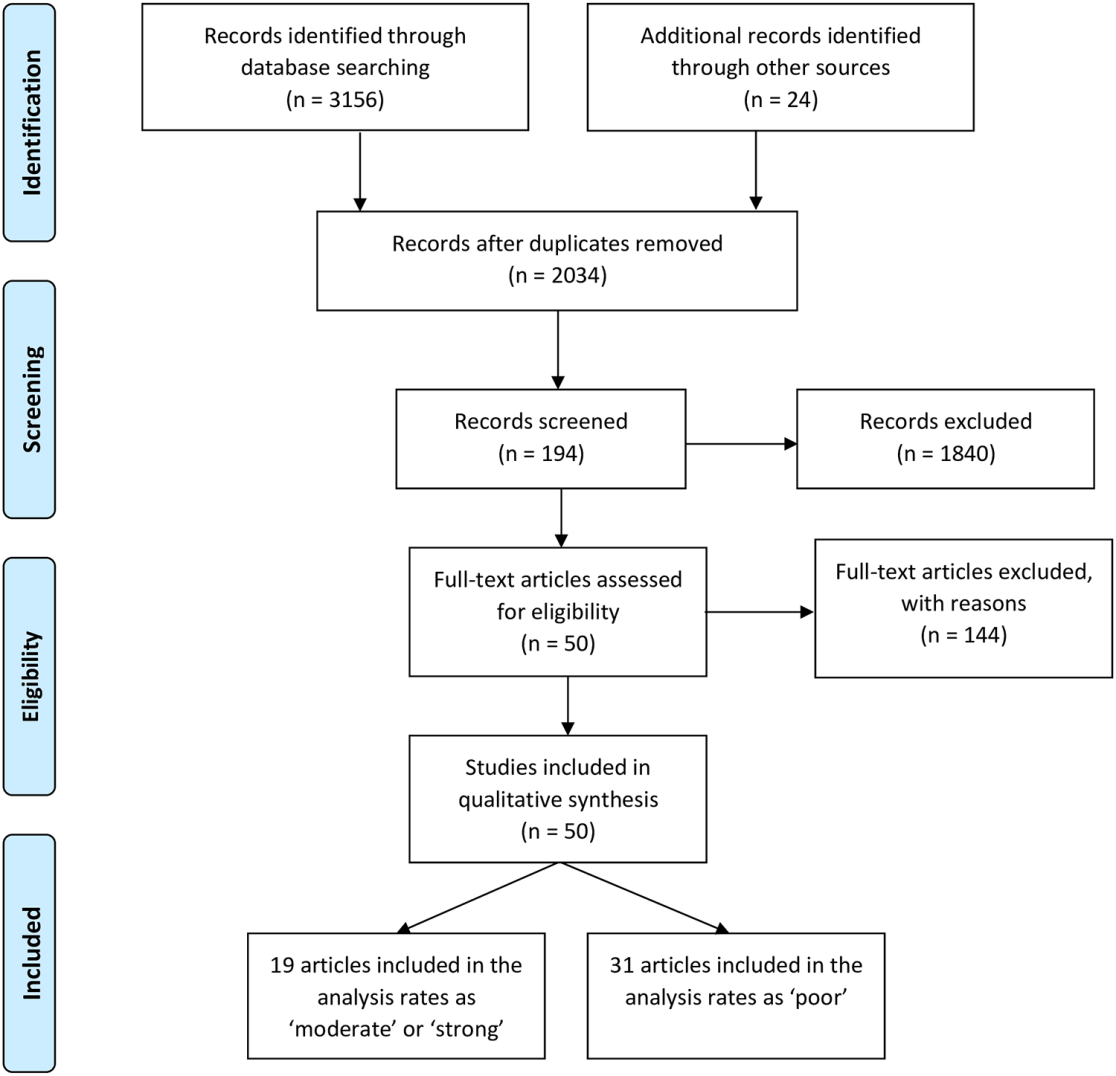
Selection process of articles from database and hand-searching following PRISMA guidelines.

The study selection followed the PRISMA guidelines [28]. The total number of articles from the database developed through the search process, and then through review of these articles reference lists were 2,034 articles excluding duplicates. The screening of these identified first round articles used eligible keywords found in the title or abstract, and resulted in 194 eligible second round articles. Exclusion criteria used in this review if they: were non-peer reviewed article, had not declared the ethical clearance process, were either review papers or opinion articles. Articles that did not present the monitoring and evaluation results were also excluded. The search process is shown in the Fig 1.

### Quality assessment and analysis

Two reviewers read 194 full-text articles independently and assessed the articles using exclusion criteria and rated included articles based on the Cochrane Handbook. Discordant results between the two reviewers were discussed with the senior researcher in the team.

The rating was based on quality relating to study design, data analysis and reporting and categorized as ‘poor’, ‘moderate’, or ‘strong’ [29]. Based on this ranking 10 articles were rated as ‘strong’, nine articles as ‘moderate’ and 31 articles as ‘poor’. Assessment the risks of bias for quantitative studies were reported in S1 Table.

Data extraction was conducted using agreed criteria developed by the team, using a form created in Microsoft Excel to assess information such as the VBDs addressed, study design, sample, data collection, analysis method, findings, reporting, discussion and ethics. A significant and non-significant outcome was reported in narrative. The analysis of data of all studies included in this review used pre-determined coding that was developed after preliminary reading of the abstracts. The code was adjusted to accommodate emergent themes with the reviewer’s concurrence. Type of intervention, sectors involved in collaboration, roles of each sectors, possible success or barrier factors related to the collaboration were identified from the studies and imported to the code list. Two reviewers conducted the coding independently, any discordance of coding results was then discussed with the senior researcher to reach a common agreement. Given the fact that only three studies measured indicators of intersectoral collaboration, the results cannot be directly compared and meta-analysis cannot be conducted across all identified articles. A conceptual framework for intersectoral collaboration was designed based on the evidence extracted from the reviewed articles.

## Results

### Characteristic of articles

S2 Table shows the characteristics of the studies included in the analysis by type of disease, study site, study design, study size and outcomes. A total of 50 studies have been included in the analysis: six articles from Region of the Americas (AMR), nine studies from Western Pacific Region (WPR), 18 studies from South East Asia Region (SEAR), 16 studies from Africa Region (AFR), and one study from AFR+AMR. Most studies discussed malaria (24 articles) and dengue (13 articles), followed by schistosomiasis (three articles), onchocerciasis (two articles), and one study for each of the following diseases: zika, leishmaniasis, lymphatic filariasis, Chagas disease, and combinations of zika + dengue + chikungunya, dengue + Japanese encephalitis, schistosomiasis + soil transmitted helminths, malaria + dengue. Of the 50 studies in the review, there were included 21 interventional studies (13 randomised control trials [RCT]; three CBA; two time series; and two uncontrolled before-after), and 29 observational studies (25 case study, four cross sectional).

Eighteen studies were conducted in rural settings, fourteen were in urban, and eight studies were taken place both in rural and urban settings, four studies were conducted in province or nationwide and nine studies did not mention the location of the study. Almost of all studies on malaria, schistosomiasis, onchocerciasis, leishmaniasis were conducted in rural areas (20 articles), while the studies on dengue, zika, chikungunya were conducted in an urban setting. The selection of the study location by the researchers was usually based on the burden of VBDs relating to suitable environment situation/s, geographic feature/s, climate condition/s and cultural landscape/s.

Exhaustive content analysis from 50 articles included in the review resulted in several themes. The themes are outcome of ISC, measuring ISC indicators, intervention in the collaboration, type of agencies involved and roles in the collaboration, and success factors and identified barriers in the collaboration.

### Excluded studies

There were 194 full text studies assessed, and 144 studies were excluded due to one or more following reasons, as shown in S3 Table:

- Review or opinion articles (n = 55)
- Did not present nor mention intersectoral collaboration (n = 58)
- Did not present evaluation data or baseline data (n = 32)
- Did not discuss an intervention for VBDs control and prevention (n = 7)
- Presented solely health sector intervention (n = 3)
- Reported community participation without intersectoral collaboration (n = 15)
- Study protocol or no ethical clearance (n = 4)

### Outcome intersectoral collaboration

Most studies reported more than one outcome as shown in S4 and S5 Tables. Of the 25 studies involving intersectoral collaboration which measured pre-post-outcomes compared against disease indicators, such as the number of cases or incidence or prevalence of malaria (15 studies) [4, 30–43], dengue (four studies) [44–47], schistosomiasis (two studies) [48, 49], zika (one study) [50], Japanese encephalitis (one study) [51], and leishmaniasis (one study) [52]. Amongst the 16 studies which measured vector variables (adult density, pupae or larval indices) as outcomes eleven studies were dengue [44, 46, 47, 53–60], followed by two studies on schistosomiasis [48, 49], and one on malaria [4] and on Chagas diseases [61].

Of the 25 studies measuring disease indicators, 22 studies reported positive effects of the intervention such as reduction of cases, and there were three studies that reported an increase in cases during the study. One case study in a post emergency situation in Philippines reported a 61% increased number of dengue cases in 2014 compared to 2013 (before a Typhoon disaster). Though an increased number of cases occurred, it did not exceed the epidemic threshold due to good collaboration between health authorities with military, NGOs, national and international donors to contain further dengue transmission in affected areas [46]. One study in Taiwan also proved the benefit of collaboration among health quarantine, immigration, and tourism industry (travel agents and tourist guides) to provide health education on dengue and zika for tourists and passengers. As a result, this collaboration succeeding in finding five zika cases during the airport screening [50].

Another case study conducted in Purworejo District, Indonesia reviewed retrospective data from 2007 to 2011. Interesting findings were that when malaria was no longer seen as a burden, the local government deducted budget allocation for malaria program and support from community and other sectors and donation from international initiative also declined, resulting in a resurgence of malaria cases from 0.56 in 2007 to 1.37 per 1000 population in 2011. Interestingly, this case study compared Purworejo District with Wonosobo. Wonosobo District succeeded in reducing malaria incidence from 0,16 in 2007 to 0.01 in 2011, although the local government cut the funding for malaria program from USD 14,699 in 2007 to USD 977 in 2011. The district health office (DHO) of Wonosobo undertook continued advocacy and negotiation with executive and legislative authorities as well as other sectors to maintain the support from community and to leverage other sectors’ funds for malaria control [38].

Similar findings were shown in vector outcomes, with almost all studies reported declining vectorial indices after the intervention. Only one study of dengue in Sri Lanka found no significant difference (p = 0.067) in pupae per 100-person index between the intervention and control groups [54]. Another study of integrated vector management (IVM) activities conducted in Malindi and Nyabondo, Kenya showed an increase of *An*. *Gambiae* and other vectors in Nyabondo, while in Malindi showed the opposite result. Beside the vector indicator, this case study also qualitatively evaluated the participation of community and intersectoral support. In Malindi that is in an urban and peri-urban area, a positive impact from IVM was found because the community who were involved in vector control succeeded in deploying to other jobs and could generate income from mosquito control such as making baskets and other household products from disposed plastic. In contrast what occurred in Nyabondo, a rural area, community participation to support malaria control program disappeared because the community did not see benefit from the project and asked for financial compensation when involved in the project activities. In this site, intersectoral collaboration was not strong as in Malindi, as the collaboration was only between MoH, Ministry of Fishery and Ministry of Education without consolidating with local authorities and NGOs to support the project [4].

Despite disease and vector outcomes, other studies also measured knowledge and behaviour change indicators, coverage of intervention, cost and policy adaptation. Nine studies that have measured an outcome of knowledge and behaviour change reflected improvement in these indicators in the intervention areas [53, 56, 59, 61–65]. All of eight studies which measured coverage of the intervention reported positive results. Four studies indicated the increased ownership and utilization of bed nets [35, 66–68]. Three studies showed improvement diagnosis and treatment of malaria [69–71], and one study found an increased number of people treated by ivermectin for onchocerciasis [72].

In term of cost analysis, one study of malaria in Ethiopia compared cost data for community- based intersectoral IRS and traditional district “vertical” IRS [73]. This study did before-after analysis of overall cost, coverage, cost per structure sprayed, and cost per person protected. In CIB-IRS district reported lower cost per structure sprayed (USD 2.27 vs 2.47 in 2013 and USD 1.98 vs 2.47 in 2014) and cost per person protected (USD 0.87 vs 1 in 2013 and USD 0.86 vs 1.01 in 2014). One evaluation study of a national campaign distributing LLINs in Tanzania calculated the cost per LLIN delivered was about USD 5.30 including production, transport and campaign costs. This cost was overall cost contributed from all parties in the campaign, and no comparison was made with LLIN distribution schemes through other sector/s [68].

### Measuring intersectoral collaboration indicators

Of 50 studies analysed in this review as shown in S4 Table, there were only three studies that measured the intersectoral collaboration in VBDs program [55, 74, 75]. No study undertook a component analysis (for different sectors or different activities). This type of study was specifically looked for this as often funders and those involved in scaling up want to understand if they need the whole package that was trialled in the “pilot’ or intervention research or if they can leave out some parts as they did not add much value. They also want to understand what might be context specific and therefore needs adjustment in other locations, and what might be universal. Only through component analysis can this be identified.

One study in Ghana measured the level of intersectoral integration in malaria control and factors associated with differentials in the levels of intersectoral integration. By using scoring interview results from 32 different institutions to calculate the level of integration, the findings revealed that level of disparity among institutions, the type of institution, level of focus on malaria and source of funding, district effect (rural vs urban) were important predictors associated with the level of intersectoral integration [74].

Another study reported on a design that compared interventions with and without intersectoral collaboration and community participation for dengue control in Cuba. In the intervention area, the mean score of participation increased and the Breteau index was lower in those communities. Furthermore, participation was also measured by indicators around participation in execution, decision making, and/or evaluation in the program. For all these indicators it found that in areas where there was intersectoral collaboration plus community empowerment the highest scores were obtained, compared to areas with only intersectoral collaboration and those without both approaches [55].

One study evaluated an international partnership that involved many countries from two regions (Africa and America), international agencies such as WHO, CDC, World Bank, and a private pharmacy company (Merck), joined together to support an onchocerciasis control program. This study measured governance and management characteristics of internal partnerships, as well as benefits and problems with the partnership by conducting semi-structure interviews and use of a self-administered survey for all people and organizations involved in the partnership. The results indicated high ratings on governance and management (such as the partnership is able to clearly define roles and responsibilities for each institution and able to implement the program and accomplish the common objectives) [75] were positive for ISC and overall VBDs outcome.

#### Interventions and level of the collaboration

Of the 50 articles, intersectoral collaboration with an intervention to mobilize community and health education was the most common. The interventions implemented through the collaboration between the sectors varied from providing human or financial resources, designing to executing an intervention, developing policy and mobilizing communities (24 studies) [30, 32, 35, 43–45, 47–50, 53–58, 60–65, 68, 72], followed by diagnosis and treatment (12 studies) [35, 36, 39–43, 49, 52, 68–71], prevention method (IRS and LLINs distribution (11 studies) [31, 35–37, 39, 41, 43, 66–68, 73], surveillance, monitoring and evaluation (nine studies) [35, 38–42, 52, 68, 69], mass drug administration (four studies) [48, 72, 75, 76], cross border collaboration (four studies) [35, 40, 43, 52], two studies for research [40, 51] and one study for advocacy and legislation [31].

The level of collaboration also varied from local, province, national, regional and global level. The level of collaboration at local level was presented by 37 studies, province or sub-national level in four studies [34, 39, 42, 51], national level in four studies [31, 50, 52, 77], regional and global level in six studies [35, 40, 43, 72, 75, 76].

### Type of agencies involved and their roles in the collaboration

There was a broad range of sectors involved in VBDs prevention and control, and in total 26 sectors involved in VBDs, see in S6 Table. These sectors included: inter-ministerial bodies such as education, parliament, defence/military, housing, immigration, agriculture, child and women welfare, rural development, fishery, public security, civil registration, data and information department, and military. In addition to inter-ministerial bodies, private sectors, central and local authorities, community members (from leaders, volunteers, forum, farmers, religious leaders), NGOs, Red Cross, university, research institutions and international agencies had been also engaged in vector borne diseases control and prevention activities.

All 26 sectors have played important and specific roles. There were nine roles and responsibilities of sectors from providing access, technical support, financial support, social mobilization, policy development, planning, implementation of activities, providing logistic, and monitoring and evaluation. This review found that roles for health sector have been identified in all of these nine roles and responsibilities. The education sector also have important roles in providing access to use school-based health program to deliver VBDs program such as utilizing teachers to provide diagnosis and treatment [48, 49, 70], teach educational material to the students [45, 48, 64, 65], and involving teacher, student, and parent association for VDBs campaign [45, 47, 49, 51, 53, 54, 58, 59, 65]. The roles of the agricultural sector identified in the studies were providing technical assistance and materials for orchard and compost production [61], land reclamation e.g. draining and filling in [4], and technical support for environmental management through introducing new farming method of applying wet and dry crop rotation that can benefit for reducing population of *Anopheles* mosquito [34].

The military also played roles in VBDs, particularly in emergency response to prevent outbreak post disaster. The study in Philippines showed positive contribution of military in preventing dengue outbreak after Typhoon [46]. In addition to emergency response, the military has a contribution in routine diagnosis and treatment, through their hospital and clinics that can serve armies and communities [41]. The military also have research units that have played essential roles in research project of VBDs vaccine, diagnosis and medicines [51].

Sharing financial resources can be one of the roles of intersectoral collaboration. The education sector can allocate budget allocation for the activities at school level [45], The agricultural sector provided budget for land reclamation [4], and plantation [61]. Public works provided financial resources to collect solid waste [56]. Central and local authorities also allocated budget for implementation of activities [4, 31, 34, 55]. Additionally, inter-ministerial bodies and government, private companies, international agencies and external expert either universities or research institutions have also contributed to finance VDBs control and prevention activities. One study of dengue control in India noted how a bank provided a loan for women’s self-help group (SHG) assigned as dengue focal point in order to generate income and able to carry social and economic activities to mobilize members and communities for VBD control [53]. Moreover, financial support also received from pharmacy companies [52, 71, 72, 75, 76], plantation companies [39, 66], mining [37] and local manufacture company [63].

Outside the health sector, social mobilization’s role was predominantly supported by education sector (teachers and students) [36, 45, 47, 49, 53, 54, 56, 62, 64, 65, 77, 78]; communities such as village leaders [41, 44, 57, 61, 78], religious leaders [54, 62], volunteers and householders [32, 36–38, 40, 41, 47, 49, 54, 56, 57, 59, 60, 63, 68], farmers [34], and community forum [38, 55, 56, 78]; community-based organization such as women group [53, 59, 76]; and media [4, 32, 62, 68].

The contribution of NGOs, either national or international organization, were not only to provide technical [33, 35, 43, 48, 61, 63, 69], financial [33, 41, 67, 72, 75] or logistics support [31, 33, 35, 43, 48, 68, 72, 75] but also to mobilize the community [31, 33, 35, 43, 48, 68, 72, 75], be involved in planning [31, 33, 35, 43, 48, 67, 69, 72, 75], implementation of activities [33, 35, 41, 43, 46, 48, 59, 67, 69] and monitoring and evaluation [33, 63, 68, 69, 72].

Historically, a VBD program was managed vertically from central government, but in this review showed that the roles of local authorities cannot be neglected. A large range of roles can be undertaken by the local authorities such as providing access for intervention [4, 35, 41, 44, 51, 53, 56, 57, 65, 77], technical support [31, 34, 55, 56], financial support [32, 35, 38, 41, 54–57, 77], social mobilization [31, 32, 56], policy development [4, 34, 35, 38, 41, 54, 56], planning [44, 54, 57, 77], providing logistics [44, 54, 57, 77], implementing the activities [36, 56, 77], and monitoring the activities [34, 55, 56, 77], see in “S4 Table”.

### Success factors and identified barriers in the collaboration

Tables 1 and 2 presents success factors and barriers identified from the evidence (detail in S7 and S8 Tables). There were six factors found to influence the success of intersectoral collaboration extracted from 47 studies. The factors discussed in the most studies were the “approach to ISC factor” (37/47) that consisted of use of a participatory approach (45%), empowering the community (45%), use of pre-existing organizations or stakeholders (43%), engagement school teachers and pupils (30%), and use socio-cultural approach (15%). The next important factors were resources (34/47), relationship (33/47), management (29/47) and shared vision (20/47) factors. Interestingly although the “type of organization” was not major success factor identified, in the one study (2%) that reported it as an important predictor for successful ISC, they found a significantly higher likelihood of non-governmental institutions integrating (Odds ratio of 11.4) than government institutions [71].

**Table 1:** Factors influencing success of intersectoral collaboration.

Influencing factors	Component of factors	Percentage of papers (n = 47)
Shared vision	Clear agreement on outcomes	43%
	Proven benefit for each sector	6%
	Complete neutrality	2%
Management	Strong management and implementation capacity	26%
	Joint planning and design program	26%
	Established committee/working group	21%
	Close supervision	21%
	Strong leadership	17%
Relationship	Consistent communication	43%
	Consistent commitments	38%
	Regular meeting	21%
	Good relationship of trust and cooperation	19%
	Open and transparent	4%
Approach	Use participatory approach	45%
	Empowered community	45%
	Use pre-existing organizations or stakeholders	43%
	Engagement school teachers and pupils	30%
	Use socio-cultural approach	15%
Resources	Received technical and financial support from international agencies	47%
	Source of funds	28%
	Legislation/policy/local system & norms	26%
	Sufficient local funds	19%
	Stable political situation	15%
Type of organizations	Government vs non-government	2%

**Table 2:** Barrier to success of intersectoral collaboration.

Barrier identified	Percentage of study (n = 21)
Political differences, Political will	57%
Poor communication and coordination	43%
Financial constraints	33%
Lack of local commitment	29%
Insufficient and irregular supplies	24%
Do not see tangible benefits	19%
Weak monitoring and evaluation	14%
Different geographic areas	10%
Professional attitudes and behaviours	10%
Inaccessible area	10%
Poor leadership	10%
The difficulties of shared decision making and power	5%
Organisational cultures	5%
Different organisational histories	5%
Organisational rigidities	5%
Contested planning priorities	5%

In the resources factor, having received technical and financial support from international agencies (47%) was the component that was the most highlighted in the studies. While in the relationship factor, consistent communication (43%) and commitments (38%) were the predominant components influencing to the success of ISC. A clear agreement on outcomes component (43%) was the most discussed in the studies as part of shared vision factor, whereas two other components were limited found in the articles: proven benefit for each sector (6%) and complete neutrality (2%). The study that raised the issue of complete neutrality as an essential factor for achieving the objectives was conducted by NGOs in cross border China-Myanmar setting [35].

Table 2 shows 16 barrier factors (in 21 studies), with political differences or political will discussed in almost of all studies (57%), followed by poor communication and coordination (43%), financial constraints (33%), and lack of local commitment (29%). Identifying a barrier to success did not mean that the project failed. When the project can overcome the barrier, the objectives can still be achieved (detail in S8 Table). For example, the study of a dengue control program in Thailand that used primary school students also identified the main constraints of collaboration among health and education sectors during the implementation of the project. Low frequency and non-regularity of the teaching and learning process, lack of consistent supervision from health staff, poor communication and coordination between different organizational personnel and insufficient, irregular and delayed supplies for school were identified as the barrier to success for the project. Learning from the process and challenges, the project was able to reduce morbidity due to dengue among schoolchildren by 219.2/100 000 pop within a period of four years [45].

## Discussion

Based on existing knowledge, this atypical systematic review of ISC in VBDs control and prevention is the first review conducted on this topic. Several systematic reviews were undertaken to measure ISC but for different issues such as health equity and primary health care.

In general, this review revealed that the benefits of and mechanisms for sectors to work together are very complex. There were many success stories of ISC on health issues in local, national and global level. In this review found consistent results that ISC on VBDs was also performed from local to global level, particularly collaboration in local level succeed when actively involving local authorities from beginning of the process. This is consistent with WHO initiative on ISC action to promote local government in health issues [79].

The range of sectors involved in VBDs intervention appears to depend on the needs in the ground. No fixed formula can suggest which sector is more effective than others and it is extremely contextual in societal, political, and geographic terms. In addition to the important roles that were played by government inter-ministerial departments, the roles of non-government organizations such as civil society, private sectors, community and religious leaders and international agencies have been found in the evidence. The issues of involving private sectors, civil society, non-government organizations and community have also been discussed in the ISC action conference at 1997 [21], where they noted the importance of improving partnerships with private sectors, NGOs, volunteers, including CBOs. The rationale is that independent of the type of project, the ultimate success of the project will depend upon participation of and commitments from individuals and community. The increased evidence on the level of and success resultant from the significant contribution of private sectors such as the nutrition industry and NGOs was also acknowledged [21, 80, 81].

This review supports the needs to collect data on ISC in VBDs control and prevention taking into account indicators of effectiveness of the ISC. This will then support an assessment of whether ISC is working or successful in achieving outcomes, and the level of contribution of ISC to that success. The review cannot comment on the level of contribution of various sectors specifically to outcome/s as no study reviewed specifically undertook a component analysis of each sector’s contribution. It is concerning that only three studies measured the indicators of ISC with a different method of analysis. Two studies [74, 75] used qualitative methods, and one study [55] used quantitative methods with RCT design to compare areas with and without ISC involvement and/or ISC involvement plus community participation. So, based on the evidence from this review we cannot make recommendations on indicators of ISC that **best fit** these approaches although some examples of what have been used have been identified [74]. However, there is framework for the vector control aspect of monitoring and evaluation ISC in IVM that can be use incorporated into the evaluation of ISC in broader VBDs prevention and control [82].

The influencing factors of the success or barrier to the success of ISC have been suggested in several studies [21, 24, 83, 84]. Some of factors were consistent with the findings in this review such as management, approach, relationship, resources and shared visions. This review then classified six major factors influencing the success of ISC on VBDs control and prevention with 25 components of factors that were found from 47 papers. Although eight studies reported that strong leadership as one of the management factors that has played a role in the achievement of effective collaboration [36, 39, 40, 52, 54, 68, 75, 76], this review found that it less important as a factor compared with the approach factors such as use pre-existing organization or stakeholders, use participatory approach as well as resources factors such as received technical and financial support from international agencies and consistent commitment and communication. Though, receiving technical and financial support from international agencies may be biased as the project have had to publish the findings as part of contract. Achieving a high level of integration requires sharing information, ideas, joint planning or modifying the delivery of services based on mutual consent from all partners [24]. Therefore, maintaining consistent levels of commitment and communication play an important role in achieving the success from ISC of IVM more so than only strong leadership factor. Use of participatory approach and empowered community were also identified as essential factors to achieve the target, it consistent with the fact that almost of VBDs control intervention requires community participation [23, 85].

Moreover, some the barriers to success identified in this review were also similar to other studies such as political differences, financial constraints, poor communication and lack of commitment [83, 84]. These factors can be used to support planning and monitoring/evaluation of ISC on VBDs in the future.

Furthermore, this review also found integrated interventions for more than one disease e.g. zika, dengue and chikungunya [60] or schistosomiasis with soil transmitted helminths [48], and integration of malaria with dengue [36]. The integrated intervention is consistent with the recent initiative to be more integrated across VBDs control and prevention [8, 10], particularly all diseases required ISC to ensure the sustainability of interventions.

There are limitations in this review. Most of the studies did not specifically measure the contribution of ISC as a specific variable, neither as outcome nor input, making it difficult to measure the contribution to other VBD outcomes. This review also cannot define the level of sustainability of IVM that can be achieved by using ISC. The search strategy was restricted to English and published peer reviewed research literature therefore many project and technical reports, and reports from non-English literature especially Spanish and Chinese are missing—potentially creating selection bias. Although use of carefully defined themes and multiple reviewers were used for the content analysis of qualitative studies there may be some reviewer bias in the review.

**Fig 2:**
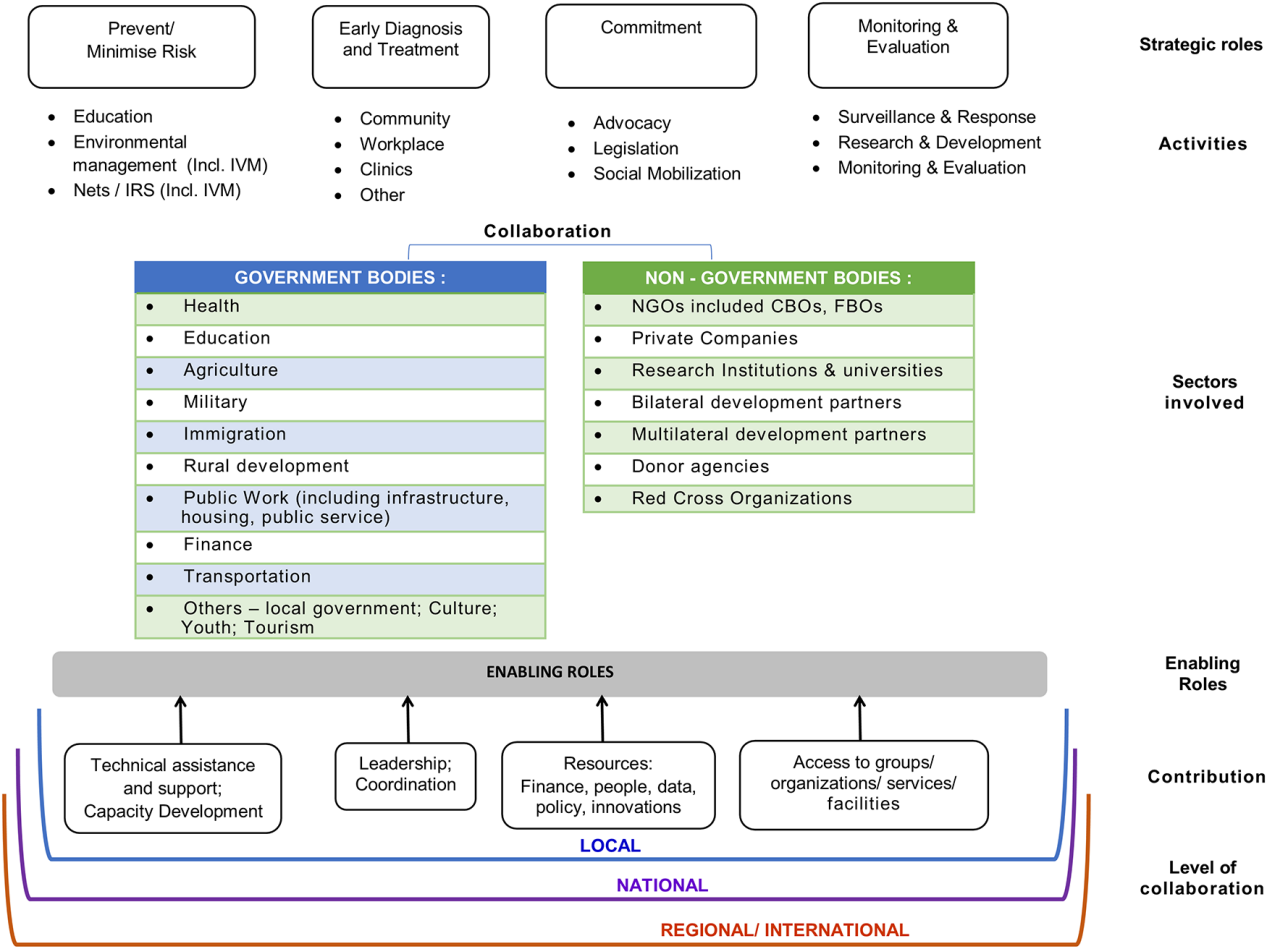
Conceptual framework of intersectoral collaboration at VBDs control and prevention based upon systematic review of peer review literature.

Based on these findings, a conceptual framework for intersectoral collaboration (Fig 2) has been developed as complement to the WHO framework of Integrated Vector Management [8]. The conceptual framework divided into six layers the core components, namely: strategic roles, kind of activities, sector involved, enabling roles, contribution, and level of collaboration. There are four strategic roles identified that can be played by each sector are 1). Prevent or minimise risk, 2). Provide early diagnosis and treatment, 3). Provide commitment, and 4). Undertake monitoring and evaluation. Each strategic role is then followed by activities. In strategic role 1, activities that aim to prevent/minimise risk of VBDs such as a) providing education to population on prevention activities, b) conducting environmental management (included IVM) that can change environment condition supported VBDs control program, and c) providing proven VBDs control intervention (included IVM) such as insecticide nets or IRS. In strategic role 2, activities that aim to provide early diagnosis and treatment can take place at community, workplaces, clinics and other locations, and through collaboration between public and private as well as involvement of community. For strategic role 3, providing commitment, advocacy, legislation and social mobilization are examples that can involve both government and non-government bodies. Strategic role 4, monitoring and evaluation, includes activities such as surveillance and response, research and monitoring and evaluation.

The framework identified two types of sectors that can work together to perform the activities supporting VBDs control and prevention, namely government and non-governmental. These sectors can collaborate within inter-government bodies or between government and non-government bodies or among non-government bodies. Flexibility in ways of implementing collaboration among sectors is required to be more effective, efficient and adaptable with the requirement for the task at hand. Four major contributions can be provided by sectors to enable the collaboration condition namely 1) technical assistance and support including capacity building, 2) leadership and coordination, 3) providing resources e.g financial, people, data, policy, innovations, and 4) facilitating access to group or organizations or services or facilities. The contribution from sectors can be based upon the requirements or functions. Finally, in VBDs programmes collaboration can be undertaken in the local, national or regional/international level according to the level of burden and scope of interventions. This framework can be used to guide a country in response to the VBDs problem with ISC approach.

It can also support a program logic for designing program especially of prevention and control of VBDs. In broader context, this framework can support the implementation the pillar of action reflected in the Global Vector Control Response [1].

## Conclusions

The recent global strategy of vector borne diseases control and prevention encourages inter-sectoral collaboration as an approach to achieve cost effective and efficient results from an intervention. This review shows inter-sectoral collaboration has played important role in achieving the impact on reduction of VBDs or vector densities. However, very few studies measured how much inter-sectoral collaboration contributed to the impact, nor for how long the ISC is needed to achieve sustainable IVM. It is recommended that further high-quality studies are undertaken using measures of indicators for inter-sectoral collaboration to address this critical gap. A conceptual framework and findings resulted in the review can be used as guideline to establish ISC on VBDs control and prevention. This work can help implementation of several recommended strategies in the Global Vector Control Response through providing an evidence base for implementers, providing some program logic to define outcomes of ISC and identifies a body of research to address gaps in robust evidence.

## Supporting information

S1 Appendix PRISMA checklist. (PDF)

S1 Table (a) Risk of bias assessment for studies included in the quantitative analysis intervention studies ((RCT and CBA), n = 17)/ (b) Risk of bias assessment for studies included in the quantitative analysis intervention studies (ITS, n = 2). (PDF)

S2 Table Characteristics of study included in the review. (PDF)

S3 Table Reasons of exclusion studies. (PDF)

S4 Table Intervention, type of sector involved and results of the study. (PDF)

S5 Table Pre and post intervention from intervention studies (n = 21). (PDF)

S6 Table Roles of multi-sectoral organization in VBDs control and prevention. (PDF)

S7 Table Factors influencing success of intersectoral collaboration. (PDF)

S8 Table Barrier to success of intersectoral collaboration. (PDF)

## Abbreviations

AFR: Africa Regional Office
CBA: Controlled Before-After
CDC: Communicable Disease Control
CIB: Community-intersectoral based
CSOs: Civil Society Organizations
DHO: District Health Office
IAH: Intersectoral action for health
IRS: Indoor residual spray
ISA: Intersectoral action
ISC: Intersectoral collaboration
IVM: Integrated Vector Management
LLINs: Long-lasting, insecticidal nets
MoH: Ministry of Health
NGOs: Non-government organizations
PAR: Pan American Region
PHC: Primary health centre
RCT: Randomized Cluster/Controlled Trial
SEAR: South East Asia Region
SHG: Self-help Group
VBDs: Vector Borne Diseases
WHO: World Health Organization
WPR: Western Pacific Region
