# Supplementary material for: Intersectoral collaboration for the prevention and control of vector borne diseases to support the implementation of a global strategy: A systematic review
Source: PLoS One. 2018 Oct 10;13(10):e0204659. doi: 10.1371/journal.pone.0204659 (PMC6179246; doi:10.1371/journal.pone.0204659)
Supplement: S1 Table — (PDF) [file pone.0204659.s002.pdf]

S1 Table. (a) Risk of bias assessment for studies included in the quantitative analysis (intervention studies (RCT and CBA), n=17)

Author/s, year	Random sequence generation (selection bias)	Allocation concealment (selection bias)	Blinding of outcome assessment (detection bias) All outcomes	Blinding of participants and personnel (performance bias) All outcomes	Incomplete outcome data (attrition bias) All outcomes	Selective reporting (reporting bias)	Baseline characteristics	Contamination	Incorrect analysis	Other bias
Abeyewickreme, W., et al, 2012	Low	Unclear	Moderate	Low	Low	Moderate	Moderate	High	Low	High
	Study area randomly selected using simple random numbers after stratified into high and low transmission sites.	Not described	Entomology outcome had assessed before and after intervention, but possible to be blinded assessment	Participants and personnel randomly selected by mapping of 200 households in the cluster	Data completed from 1600 HH	Secondary findings were not reported in detail	Reported and borderline statistically significant different	Similar access to information	Adjusted for baseline characteristics	Risk of confounding
Afenyadu, G.Y., et al., 2005	Low	Unclear	High	Low	Low	Low	Unclear	Low	High	High
	Intervention randomly allocated	Not described either teacher or investigator could see the treatment package	No baseline measurement of outcome	Participant randomly selected for outcome assessment.	Low missing data	All findings were reported	No information reported	Unlikely that the control group received the intervention	Not adjusted for clustering	Risk of confounding
Arunachalam et al, 2012	Low	Unclear	Moderate	Low	Low	Low	Low	Low	High	High
	Study area randomly selected using simple random numbers after stratified into high and low transmission sites.	Not described	KAP and entomology outcome had assessed before and after intervention, but possible to be blinded assessment	Participants and personnel randomly selected by mapping of 100 households in the cluster	Data completed from 2000 HH	All findings were reported	Reported and similar	Distance among cluster was approximately 180 squares	Not adjusted for clustering	Risk of confounding

Author/s, year	Random sequence generation (selection bias)	Allocation concealment (selection bias)	Blinding of outcome assessment (detection bias) All outcomes	Blinding of participants and personnel (performance bias) All outcomes	Incomplete outcome data (attrition bias) All outcomes	Selective reporting (reporting bias)	Baseline characteristics	Contamination	Incorrect analysis	Other bias
De Urioste-Stone, S.M., et al., 2015	Low	Unclear	Moderate	Moderate	Low	Low	Low	Unclear	High	High
	Study area randomly selected using a probability systematic sampling design	Not described	KAP and entomology outcome had assessed before and after intervention, but possible to be blinded assessment	Participants and housed from selected clusters were included as samples.	Data completed	All findings were reported	Reported and similar	Not stated	Not adjusted for clustering	Risk of confoun ding
Deribew, A., et al., 2012	Low	Unclear	High	Low	Low	Low	Low	Unclear	Low	Low
	Study area randomly selected.	Not described	Primary outcome were not screened blinded.	Participant randomly selected for outcome assessment.	Data completed	All findings were reported	Reported and similar	Not stated	Adjusted for clustering	Adjusted for confoun ding (sex and age)
Johns, B., et al., 2016	Moderate	Low	Low	Low	Low	Low	High	Low	High	Unclear
	Study area selected purposely from intervention and control area. Evaluation selected randomly.	Unit of allocation was performed on all units at the start of the study	Primary outcome were measured blinded	Participant randomly selected for outcome assessment.	Data completed	All findings were reported	Some missing baseline data from control	Unlikely that the control group received the intervention	No adjusted for cluster	Not describe d

Author/s, year	Random sequence generation (selection bias)	Allocation concealment (selection bias)	Blinding of outcome assessment (detection bias) All outcomes	Blinding of participants and personnel (performance bias) All outcomes	Incomplete outcome data (attrition bias) All outcomes	Selective reporting (reporting bias)	Baseline characteristics	Contamination	Incorrect analysis	Other bias
Kittayapong, P., et al., 2006	Unclear	High	High	High	Low	Low	Unclear	High	Unclear	High
	Not stated	Controlled before and after study	Primary outcome were not screened blinded.	All community and houses included outcome assessment.	Data completed	All findings were reported	Not presented baseline characteristics.	Distance intervention only 100 meter.	Cluster adjustment not applicable	Not adjusted for confounding
Kittayapong, P., et al., 2012	Low	High	High	High	Low	Low	Low	Unclear	Unclear	High
	Cluster was randomly selected.	Key stakeholders were informed and involved in designing intervention tools and strategies	Primary outcome were not screened blinded.	All community and houses included outcome assessment.	Data completed	All findings were reported	Reported and similar.	Not described	Cluster adjustment not applicable	Not adjusted for confounding
Kittayapong, P., et al., 2008	Unclear	High	High	High	Low	Low	Low	Unclear	Unclear	High
	Not described.	Key stakeholders were informed intervention tools and strategies.	Primary outcome were not screened blinded.	All community and houses included outcome assessment.	Data completed	All findings were reported	Reported and similar.	Not described	Cluster adjustment not applicable	Not adjusted for confounding

Author/s, year	Random sequence generation (selection bias)	Allocation concealment (selection bias)	Blinding of outcome assessment (detection bias) All outcomes	Blinding of participants and personnel (performance bias) All outcomes	Incomplete outcome data (attrition bias) All outcomes	Selective reporting (reporting bias)	Baseline characteristics	Contamination	Incorrect analysis	Other bias
Sanchez, 2009	Moderate	High	Unclear	Moderate	Unclear	Low	Unclear	Moderate	Unclear	Unclear
	Systematic random sampling for respondents to evaluate population's involvement.	Consejo Popular in the intervention area was explained the objective of study and approved the study.	Not described	Participants was blind selected, but containers and houses was not randomly inspected	Not measure loss to follow up.	All data collected were reported	Not stated	Program was managed by higher level authorities, contamination between areas cannot be excluded	Only calculate different proportion and mean index	Not describe d
Sanchez, 2005	Moderate	High	Low	Moderate	Unclear	Low	Unclear	Moderate	Unclear	High
	Two-stage randomised sampling for KAP study	Consejo Popular in the intervention area was explained the objective of study and approved the study.	Baseline outcome was measured prior the study. All outcomes measured blinded	Participants was blind selected, but containers and houses was not randomly inspected	Individual selected was not follow up, not measure loss to follow up.	All data collected were reported	Not stated	Control areas are located side by side.	Only calculate different proportion and mean index	High of confoun ding from ecology and climate factors
Sedlmayr, R., et al.,2013	Low	Low	Low	Low	Low	Low	High	Low	Low	High
	All intervention and control area selected using stratified cluster randomized design	Unit of allocation was performed on all units at the start of the study	Baseline outcome was measured prior the study. All outcomes measured blinded	Participant randomly selected for outcome assessment based on their cluster	Data completed	All data collected were reported	Baseline characteristics was similar, but not measured the ownership and utilization before intervention	ITNs can be used by all farmers free and without conditions.	Adjusted for clustering and age	Bias from self-reported malaria morbidit y

Author/s, year	Random sequence generation (selection bias)	Allocation concealment (selection bias)	Blinding of outcome assessment (detection bias) All outcomes	Blinding of participants and personnel (performance bias) All outcomes	Incomplete outcome data (attrition bias) All outcomes	Selective reporting (reporting bias)	Baseline characteristics	Contamination	Incorrect analysis	Other bias
Tana S, et.al, 2012	Low	High	Low	Low	High	High	High	High	Unclear	Unclear
	All intervention and control area selected using randomly	Key stakeholders were informed intervention tools and strategies.	Baseline outcome was measured prior the study. All outcomes measured blinded	Participant randomly selected for outcome assessment based on their cluster	Data from control group was not presented.	Data from control group was not presented	Baseline data was not presented	Cluster extended following administrative boundaries	Analysis of vectorial indices did only for interventio n areas	Not describe d
Ulibarri, G., et al., 2016	High	Low	High	High	High	High	High	Unclear	Unclear	Unclear
	Randomisation only for vector control intervention.	Unit of allocation was performed on all units at the start of the study	Blinding outcome assessment only for Ecological ovillanta.	Blinding of participants and personnel only for ovillanta intervention.	Data from control group was not all presented	Data from control group was not all presented	Baseline data was not presented	Not described	Not completed data presented and analysis	Not describe d
Vanlerberghe, V., et al., 2009	Low	High	High	Moderate	Low	Moderate	Low	Unclear	Low	High
	Cluster was randomly selected.	Key stakeholders were informed and involved in designing intervention tools and strategies	Primary outcome were not screened blinded.	Participants was blind selected, but containers and houses was not randomly inspected	Data complete and reported	Secondary findings were not reported in detail	Reported and similar.	Not described	Cluster adjustment applicable	High of confounding from ecology and climate factors
Wai, K.T., 2012	Low	High	Low	Moderate	Low	Low	High	Unclear	High	High
	Intervention and control randomly selected	Key stakeholders were informed and involved in designing intervention tools and strategies	Baseline outcome was measured prior the study. All outcomes measured blinded	Participants and housed from selected clusters were included as samples.	Data completed	All data collected were reported	Baseline data was not separated between control and intervention areas	Not described	Not adjusted for clustering	High of confounding from ecology and climate factors

Author/s, year	Random sequence generation (selection bias)	Allocation concealment (selection bias)	Blinding of outcome assessment (detection bias) All outcomes	Blinding of participants and personnel (performance bias) All outcomes	Incomplete outcome data (attrition bias) All outcomes	Selective reporting (reporting bias)	Baseline characteristics	Contamination	Incorrect analysis	Other bias
Yuan, L.P., et al., 2005	Low	High	Low	Low	Low	High	High	Unclear	Unclear	High
	Intervention and control randomly selected	Teacher and parents were informed and involved in designing intervention tools and strategies	All outcomes assessed blinded	Participants blinding in the institutions	No missing data	Only reported post intervention results	Baseline data was not presented	Not described	Cannot compared pre and post test results	Risk of confounding

S1 Table. (b) Risk of bias assessment for studies included in the quantitative analysis (intervention studies (ITS, n=2)

Author, year	Intervention independent of other changes	Shape of the intervention effect pre-specified	Intervention unlikely to affect data collection	Knowledge of the allocated interventions adequately prevented	Incomplete outcome data adequately addressed	Selective outcome reporting	Other risks of bias
Kaatano, G.M., et al., 2015	High	Low	Low	Unclear	High	Low	High
	Intervention changed over the time	Analysis conducted after point of intervention	Sources and methods of data collection were the same before and after the intervention	Not described blinded data collection	Missing outcome data was likely to bias the results	All outcome reported	No adjustment for changed people administering drug
Magnussen, P., et al., 2001	Low	Low	Low	Unclear	Unclear	Low	High
	Intervention not changed over the time	Analysis conducted after point of intervention	Sources and methods of data collection were the same before and after the intervention	Not described blinded data collection	Not described mising data	All outcome reported	Risk of confounding environment and snail data was not measured
