# Supplementary material for: Intersectoral collaboration for the prevention and control of vector borne diseases to support the implementation of a global strategy: A systematic review
Source: PLoS One. 2018 Oct 10;13(10):e0204659. doi: 10.1371/journal.pone.0204659 (PMC6179246; doi:10.1371/journal.pone.0204659)
Supplement: S2 Table — (PDF) [file pone.0204659.s003.pdf]

S2 Table. Characteristics of study included in the review

SUMMARY OF ALL ARTICLES										
**Author/s, year**	**VBD**	**Study site/s**	**WHO Region**	**Duration of study**	**Research method/s used**	**Study design**	**Quality**	**Setting**	**Study size**	**Outcome/s included in the review**
**Intervention study**										
Abeyewickreme, W., et al, 2012	Dengue	Sri Lanka	SEARO	12 months	Mixed	RCT	Strong	Urban and peri-urban	1600 Households	Stakeholder analysis, socialogical assessment and pupae per 100 persons index (PPI)
Afenyadu, G.Y., et al., 2005	Malaria	Ghana	AFRO	Eight months	Mixed	RCT	Moderate	Rural	241 teachers	% of corrective treatment of presumptive malaria in school children
Arunachalam et al, 2012	Dengue	India	SEARO	10 months	Mixed	RCT	Strong	Urban	2000 households	Knowledge, practices and attitude of community, health care providers on dengue control. Larva and pupae index
De Urioste-Stone, S.M., et al., 2015	Chagas diseases	Guetamala	PAHO	2010 - 2014	Mixed	RCT	Strong	Urban	248 Households	PCR-based Triatomine infestation from gut tissue of first, second and third instar nymphs

									472 respondents for KAP study. In-depth interview: n=48	KAP survey pre-and post-intervention. Stakeholder analysis. Participatory research action of community
Deribew, A., et al., 2012	Malaria	Ethiopia	AFRO	12 months	Mixed	RCT	Strong	Rural	in total 7059 children under five	Prevalence of malaria and anaemia among children under five. Perception of the community about the utilization of LLINs and burden of malaria
Johns, B., et al., 2016	Malaria	Ethiopia	AFRO	2012 - 2014	Mixed	RCT	Strong	Not stated	11 districts	Coverage of IRS and cost for IRS
Kaatano, G.M., et al., 2015	Schistosomiasis and STH	Tanzania	AFRO	2009 - 2012	Quantitative	Interrupted time series (ITS)	Moderate	Rural	400 adults and 150-200 schoolchildren	Prevalence of schistosomiasis and hookworms in adult and schoolchildren.
Kittayapong, P., et al., 2006	Dengue	Thailand	SEARO	2012 - 2014	Quantitative	Controlled before-after (CBA)	Moderate	rural and semi-rural	2 villages	Larval density, proportion positive container with different methods
Kittayapong, P., et al., 2012	Dengue	Thailand	SEARO	six months	Quantitative	RCT	Strong	urban and peri-urban	All houses and public facilities in the clusters	Vector densities (HI, CI, PPI)

									320 respondents for assess acceptance	Acceptance of community on vector control measure.
Kittayapong, P., et al., 2008	Dengue	Thailand	SEARO	four months	Quantitative	RCT	Moderate	urban and peri-urban	All houses and public facilities in the clusters<br><br>6 schools, approximately 1,800 schoolchildren.	Average number of positive containers per house and the average number of pupae per house with 95% CI).<br><br>Proportion of IgG–IgM positive students.
Kusuma, Y.S., et al., 2017	Dengue	India	SEARO	2013	Mixed	Interrupted time series (ITS)	Moderate	Urban	496 households	Proportion different pre and post intervention.
Magnussen, P., et al., 2001	Schistosomiasis	Tanzania	AFRO	5 years	Quantitative	Time series design	Moderate	Rural	2379-2892 school children registered in 11 primary school<br>All children in class 5 from 11 primary schools (170-200 children)	Prevalence of haematuria, intensity of infection

Okabayashi, et al. 2006	Malaria	Thailand	SEARO	2005	Quantitative	Uncontrolled before-after	Poor	Not stated	Of these, 14 school principals, 93 teachers and 631 schoolchildren joined the study	Change in knowledge and practice of teachers and schoolchildren on malaria control
Sanchez, 2009	Dengue	Cuba	PAHO	5 years	Mixed	Controlled before-after (CBA)	Strong	Urban	125 households in the pilot and extension areas	• Breteau index (BIs, number of positive containers per 100 houses) over time periods per area and type of intervention.<br>• Mean of participation after initiating intersectoral coordination and community empowerment
Sanchez, 2005	Dengue	Cuba	PAHO	12 months	Mixed	Controlled before-after (CBA)	Moderate	Urban	125 households<br><br>Routine entomology surveillance data	• KAP differences in proportion before and after intervention.<br>• House index (HI) and Container Index (CI) before and after intervention.

Sedlmayr, R., et al.,2013	Malaria	Zambia	AFRO	6 months	Mixed	RCT	Strong	Rural	1440 farmers	• Self-reported morbidity of malaria using proxy of having fever in the last two weeks and confirmed malaria by laboratory. • Coverage of bed nets utilization
Tana S, et.al, 2012	dengue	Indonesia	SEARO	2 years	Mixed	RCT	Strong	urban	423 houses and 100 households	Increasing KAP in dengue prevention, community participation and its sustainability and ownership. Declining PPI and BI
Ulibarri, G., et al., 2016	Dengue, Chikungunya, Zika	Guatemala	PAHO	11 months	Mixed	RCT	Strong	urban	16 focus groups; 25 health workers, 84 households for ecological ovilanta	Effectiveness of the integrated intervention to control Aedes spp. Through training of health workers, use of low-cost ecological ovillanta and community engagement.
Vanlerberghe, V., et al., 2010	Dengue	Cuba	PAHO	2005-2007	Mixed	RCT	Strong	Not stated	32 circumscriptions (around 2000 inhabitants each)	House index, Breteau index, and the pupae per inhabitant statistic

Wai, K.T., 2012	Dengue	Myanmar	SEARO	Nine months	Mixed	RCT	Moderate	Urban	1200 householders	PPI (pupae per person index),
Yuan, L.P., et al., 2005	Schistosomiasis	China	WPRO	2000	Mixed	RCT	Strong	Not stated	30 schools	Change in knowledge on schistosomiasis control
Argaw, M.D., et al.,2016	Malaria	Ethiopia	AFRO	2015	Quantitative	Cross sectional (retrospective)	Poor	Rural and urban	110 private malaria facilities	Proportion malaria suspected case, malaria slide positivity rate, proportion malaria case treated by standard medicine
Aumentado, C., et al., 2015	Dengue	Philipines	WPRO	2014	Quantitative	Case study	Poor	Urban and semi-urban	8 hospitals/clinics near Tacloban City	Number of weekly dengue case, Breateu Index
Bhattacharya, S.K., and Dash, A.P., 2017	Leishmaniasis	India	SEARO	2016	Quantitative	Case study	Poor	Rural	One countries	Number of cases and number of death cases related to Leishmaniasis
Castro, M.C., et al., 2009	Malaria	Tanzania	SEARO	One year	Quantitative	Cross sectional	Poor	Urban and peri-urban	9070 respondents	Descriptive analysis on demographic characteristic, recent malaria infection, perception of presence of waste in drains, and willingness to participate in community-based EM activities.

									Routine surveillance data	Unadjusted and adjusted malaria prevalence.<br>Proportion of water habitant contained pupae and/or Anopheles larvae.
Chanda et al, 2008	Malaria	Zambia	AFRO	5 years (2003-2007)	Quantitative	Case study	Poor	unclear	N/a	Malaria incidence and mortality. Proportion of IRS and ITN.
Chandiwana, S.K., et al., 1991	Schistosomiasis	Zimbabwe	AFRO	1985-1989	Quantitative	Case study	Poor	rural	N/a	Prevalence of heavy infections (>50 eggs per 10 ml of urine) for 1985 (pre-treatment) 1986 (3 months after treatment) and 1987 (8 months after treatment).<br>Annual change in the percentage of Bulinus globosus (snail host for s. haematobium) during the peak transmission
Drameh, P.S.,et al., 2002	Onchocerciasis	19 African countries	AFRO	2001	Qualitative	Case study	Poor	Not stated	One region	Number of people treated by Ivermectin
Ghosh, S.K., et al., 2006	Malaria	India	SEARO	Des-01	Quantitative	Case study	Poor	Rural	87 households consisted of 289 people	Change in knowledge on malaria control

Gibbons, R.,V., et al., 2013	Dengeu, Japanese Ensephalitis	Thailand	SEARO	2012	Qualitative	Case study	Poor	Rural	One Province	Number of paper published in success of JE vaccine efficacy in routine program,
Herdiana, et al., 2013	Malaria	Indonesia	SEARO	2012	Quantitative	Case study	Poor	Rural	One district	Confirmed malaria case per thousand population
Ho, L.L., et al., 2017	Zika	Taiwan	WPRO	2016	Quantitative	Case study	Poor	Urban	All inbound passengers	Number of suspected DHF and Zika case confirmed by RDT
Ichimori, K., and Crump., A., 2005	Lymphatic Filariasis	Pacific	WPRO	2011	Quantitative	Case study	Poor	Urban and rural	One region	Number of countries and areas implementing MDA strategy
Kong, X., et al.,2017	Malaria	China	WPRO	2016	Quantitative	Case study	Poor	Not stated	One province	Number of malaria indigenous case
Krisher, L.K., et al., 2016	Malaria	Ecuador-Peru	PAHO	1990-2012	Quantitative	Case study	Poor	Rural	Two countries	Malaria incidence per 10,000 population
Martins, J.S. et al., 2012	Malaria	Timor Leste	WPRO	4 years (2003-2006)	Mixed	Case study	Poor	Not stated	58 key informants for in-depth interview, 8 groups interviews, 16 FGDs<br>Routine surveillance report	Opinion on contribution of global fund grant for malaria control program and health system. Malaria morbidity.

Murhandarwati, E.E., et al., 2015	Malaria	Indonesia	SEARO	2012	Mixed	Case study	Poor	Rural	One district	Number of malaria cases by year, plasmodium type, gender. Mapping of endemic villages. Coverage of malaria control intervention (diagnosis, ACT, LLINs, IRS)
Mutero, C.M., et al., 2015	Malaria	Kenya	AFRO	Not described	Mixed	Case study	Poor	Rural and urban	District population	Proportion of malaria cases recorded in health facilities, densities of mosquitoes.
Njau, R.J., et al., 2009	Malaria	Tanzania	AFRO	2005 - 2006	Mixed	Case study	Poor	Rural	24 key informants for interview, and 24 women of child-bearing age for FGDs 1,048 women of child-bearing age.	Proportion of ITN ownership among women of child-bearing age
Owusu, N.O., et al., 2013	Malaria	Ghana	AFRO	Two months	Mixed	Cross sectional	Moderate	Rural and urban	512 observations nested within 32 institutions.	Factors associated to level of integration among intersectoral.

Oyediran, et al, 2002	Malaria	Kenya, Uganda	AFRO	1999-2000	Quantitative	Case study	Moderate	Nationwide	8 pilot sites	positive effect by improving diagnosis and treatment management
Peters, D.H., and Phillips, T., 2004	Onchocerciasis	MDP secretariat	AFRO and PAHO	Not described	Mixed	Case study	Poor	Not stated	Two regions	Factors associated to sustainability of MDP program.
Qunhua, L., et al., 2004	Malaria	China	WPRO	50 years	Quantitative	Case study	Poor	Rural	N/a	Malaria incidence per 10,000 population, malaria mosquito population
Renggli, S., et al., 2013	Malaria	Tanzania	AFRO	15 months	Quantitative	Case study	Poor	Rural and urban	2059 households from seven districts.	Coverage of bed nets ownership.<br>Cost for delivery LLINs by each net
Sanders et al, 2014	Malaria	Malaysia	WPRO	2014	Qualitative	Case study	Poor	Rural	One state	Number of malaria cases in the plantation areas.
Sharp B., et al., 2002	malaria	Zambia	AFRO	2 months	Mixed	Case study	poor	Rural and urban	N/a	reduction of malaria incidence<br>Efficacy of house spraying<br>unit cost for IRS was cheaper than bednet
van den Berg, H., et al., 2012	Malaria and dengue	Philipines	WPRO	One month	Qualitative	Case study	Poor	Rural and urban	N/a	Operational management of vector control at different administrative level

Wangroongsarb, Y., 1997	Dengue	Thailand	SEARO	Three months	Quantitative	Case study	Poor	Rural and urban	371 people from 96 schools	Dengue morbidity rate
Watanabe et al, 2015	Malaria	Vanuatu	WPRO	Jul-12	Qualitative	Case study	Poor	rural	30 discussants, 10 informants, 17 interviewes	Community engagement, which facilitates local personal and social-contextual resource development, has potential for Malaria Elimination and multilevel empowerment through community-based capacity development processes.
Xu, J.W., et al., 2016	Malaria	China – Myanmar border	SEARO	Jan 2008-May 2014	Mixed	Cross Sectional	Poor	Rural	24 sites	Prevalance parasite rate for Myanmar region, API for counties in China.
Zhang., J., et al, 2016	Malaria	China – Myanmar border	SEARO	2006-2013	Quantitative	Case study	Poor	Rural	N/a	Coverage of utilization of LLIN, malaria parasite and mortality rate
