# Supplementary material for: Intersectoral collaboration for the prevention and control of vector borne diseases to support the implementation of a global strategy: A systematic review
Source: PLoS One. 2018 Oct 10;13(10):e0204659. doi: 10.1371/journal.pone.0204659 (PMC6179246; doi:10.1371/journal.pone.0204659)
Supplement: S3 Table — (PDF) [file pone.0204659.s004.pdf]

S3 Table. Reasons of exclusion studies

ID ref	Study (Name authors, year)	Reason of exclusion
2	Abuelmaali, S.A., et al, 2013	No explicit collaboration intervention stated in the study. Practice of farmer using insecticides for agriculture affected malaria mosquito resistance.
7	Bernardini Zambrini, D.A., 2011	Opinion on epidemic dengue in Argentina at 2009. No intervention presented in the articles.
9	Boussalis, C., 2012	No intersectoral collaboration measured. The study assessed modelling of sub-national government capacity to estimate malaria incidence with adjusting several extrinsic indicators.
10	Breman, J.G. and C.N. Holloway, 2007	Review article on malaria surveillance counts. No baseline data was reported
11	Brieger, W.R., 1984	Case study on collaboration between health educators and medical scientists in bridging communication with community. No evaluation data on VBDs was reported.
12	Brouqui, P., 2012	No intersectoral collaboration was measured. RCT for insecticide resistance.
13	Byron, Y., et al., 2015	Commentary. No baseline data was reported.
14	Caldas de Castro, M., et al., 2004	Case study of multiple vector control strategies of malaria. No intersectoral collaboration was presented.
15	Cancado, M.S., et al., 2014	Perception of intersectoral committee of dengue regarding health education. No intervention and evaluation were presented.
17	Chanda, E., et al., 2017	Review IVM in Africa.
18	Chanda, E., et al., 2013	Review malaria control program at Zambia. Intersectoral collaboration among different sectors, international partners, local NGOs, and community participation were recognized in the articles to reduce malaria cases. However, no specific measurement for the collaboration.
20	Chanda, E., et al., 2014	Review LLINs distribution in South Sudan 2006-2012, no intersectoral collaboration is presented.
22	Dambach, P., et al., 2016	No evaluation data of intervention was reported in the article.
23	de Paula Fonseca, E.F.B. and F. Zicker, 2016	No evaluation data of intervention was reported in the article.
26	DeRoeck, D., 2003	Opinion of policymakers on dengue vaccine.

ID ref	Study (Name authors, year)	Reason of exclusion
27	Dhariwal, A.C., et al., 2014	No evaluation data of intervention was reported in the article.
28	Dial, N.J., et al.,2014	Community acceptance on mass drug administration of malaria. No intervention and evaluation data were presented.
30	Ellis, B.R. and B.A. Wilcox, 2009	Review document of ecology of vector borne diseases research and control.
31	Fawole, O.I., et al, 2007	Case management of childhood fever by traditional healers. No intersectoral collaboration was presented
32	Fernando, D., et al, 2007	Study patient satisfaction with private health providers serviced for malaria screening and diagnosis. No intersectoral collaboration was measured.
33	Fletcher, M. et al., 1992	RCT for mosquito larvae control using an indigenous larvivorous fish. However, the intervention was conducted by health sector only, multi-sectoral collaboration was not reported.
34	Ghanekar, M.A., et al.	Multi stakeholders collaboration on Urban climate change resilience, not about VBDs
35	Ghebreyesus, T.A., et al.1996	Community participation in malaria control, not measure collaboration with other sectors
38	Gilles, H.M., 1993	A text book: essential malariology
39	Gonzalez Fernandez, M.I. et al., 2010	Qualitative policy analysis on dengue control. No evaluation data was reported.
40	Greenwood, B., A. Bhasin, and G. Targett, 2012	Gates malaria partnership supported training program. No intersectoral collaboration was measured.
41	Grepin, K.A., et al., 2014	China's role as a global health donor. No intersectoral collaboration was measured.
42	Griffiths, K., et al., 2013	Qualitative response on outbreak dengue in Nepal. No evaluation data was reported.
43	Gryseels, B., 1992	Control of Schistosomisis mansoni. Not measure collaboration with other sectors
44	Gubler, D.J., 2015	Letter to editor.
45	Gurtler, R.E., 2009	Review vector control strategies in the Gran Chaco Region. No evaluation data was presented.
46	Gurtler, R.E., 2015	Eco-bio-socio research on community based approach on Chagas disease, the role and involvement of the community and intersectoral were not presented clearly.

ID ref	Study (Name authors, year)	Reason of exclusion
47	Gutteridge, W.E., 2006	TDR collaborated with phamacy industries. No other intersectoral collaboration and evaluation data was reported.
48	Hanvoravongchai, P., 2010	Critical interaction between Global Fund and Health System, not specific for VBDs and intersectoral collaboration, only the role of the Global Fund was presented.
49	Haq, Z. et al., 2013	Review communicable diseases in the Eastern Mediterranean Region, not specific for vector borne diseases.
50	Hay, S.I., 2002	A UNICEF SSA report on Intersectoral response to malaria outbreak. No evaluation data was reported
51	Hemingway, J., 2014	Review role of vector control to stop malaria elimination. No evaluation data was presented.
54	Holveck, J.C., et al., 2007	Opinion on intersectoral approach at NTDs.
55	Hunter, J.M., I, 2003	Review the effect of uncoordinated construction of dam and its maintenance issues among outside health sectors with health sectors caused increasing schistosomiasis cases.
57	Ingabire, C.M., et al., 2014	Qualitative community perception to eliminate malaria. No evaluation data was reported.
58	Ingabire, C.M., et al., 2016	Community participation for achieving malaria elimination. No intersectoral collaboration was presented
59	Jentes, E.S., et al., 2017	Described interagency and private collaboration on screening dengue and chikungunya at Miami airport. No evaluation data was presented.
61	Jones, C.H., et al., 2014	Perspective and acceptance of community on insecticide screen as preventive tool for dengue. No intersectoral collaboration was presented.
62	Jones, J.T., et al., 2004	Review international scientific collaboration using several projects as examples.
64	Kamat, V.R, 2005	Private health practioners in malaria diagnosis and treatment. No other intersectoral collaboration was reported.
65	Kamuliwo, M., et al., 2013	Review malaria burden in Zambia from 2006 to 2011. No intersectoral collaboration was measured.
66	Kandiah, 1990	Review on the role and responsibilities of engineers and agriculturalists in reducing vector-borne disease hazards. No evaluation data was presented.
67	Kaneko, A., 2010	Community involvement for sustainable malaria elimination. No intersectoral collaboration is presented.

ID ref	Study (Name authors, year)	Reason of exclusion
68	Katz, I., et al., 2011	Projection of GFATM financial support for ARV HIV, TB cases and LLINs.
69	Kibret, S., et al, 2015	A study on malaria impact of the large dams. No intersectoral collaboration was measured
70	Kiefer, S., et al., 2017	Review GFATM financial support for implementation and operational research on TB and malaria.
71	Kitron, U., 1987	Review malaria control campaign and effect of agriculture and development.
75	Klinkenberg, E., 2004	Risk factor analysis. No intersectoral collaboration was measured and evaluated.
76	Klueh, U.H., et al., 2007	Intersectoral for oil industry, not for VBDs
77	Knopp, S., et al., 2012	A study protocol.
78	Kolaczinski, J. and J. Webster, 2003	Review malaria control in East Timor during emergencies. No outcome is reported
81	Kumar, V., et al., 2009	Only health sectors did the intervention.
83	Laktabai, J., et al., 2017	A study protocol.
84	Larrieu, S., et al., 2016	A surveillance protocol for Zika emergency preparedness.
85	Lee, M.S., et al., 2003	Review framework of monitoring malaria eradication in Korea.
86	Leontsini, E., et al., 1993	Community involvement on dengue vector control. No intersectoral collaboration is presented.
87	LI et.al., 2015	A study on intersectoral colaboration for healthy urban planning not VBDs
88	Mack, A., 1997	Opinion
90	Mahabir, R.S., 2012	Impact of road networks on the distribution of dengue cases. No intersectoral collaboration is presented
92	Mbonye, A.K. and P. Magnussen, 2013	Commentary on translating health research into policy.
93	Mgone, CS, 2010	Editorial One world One Partnership to fight HIV, TB and malaria
94	Mlozi, M.R., et al., 2015	Perception of stakeholders regarding challenges and opportunities of intersectoral collaboration for malaria control program. No evaluation data was reported.
95	Molyneux, D., 2003	Review Lymphatic Filariasis elimination progress.
96	Molyneux, D.H. and V. Nantulya, 2005	Review PPP of river blindness in distribution ivermectin drug.
97	Mons, B., et al., 1998	Review partnership on malaria between north and south. No evaluation data was presented.

ID ref	Study (Name authors, year)	Reason of exclusion
98	Moonasar, D., et al., 2012	Review malaria control program in South Africa 2000 – 2010.
99	Morris, K., 2000	Opinion on malaria control partnership.
100	Mota, F.B., et al., 2017	Mapping dengue research and network. No intersectoral collaboration is measured.
101	Mott, et. Al., 1995	Review on New geographical approaches to control of some parasitic zoonoses, not measure the intersectoral collaboration
102	Moyou-Somo, 2013	KAP study on malaria control and prevention for home-based management and prevention of childhood malaria. No intervention and evaluation data were presented.
105	Mutero, C.M., et al., 2012	Perception of stakeholders on IVM in Uganda. No evaluation data was presented
106	Muturi, EJ, et al,2008	Effect of Rice cultivation on Malaria Transmission in Central Kenya. No intersectoral collaboration was measured.
107	Mwisongo, A., et al., 2016	Perspective of global health initiative in Tanzania. No evaluation data was presented.
108	Nathan, M.B., et al, 2004	Case study community participation on dengue control in Carribean. Not evaluation data was presented.
109	Navaratnam, et al, 2012	Innovative Partnership and approach in the development of ASMQ for malaria. No evaluation data on intersectoral collaboration
110	N'Diaye, M., et al., 2016	No collaboration with other sectors was presented
111	Negev, M., et al., 2015	Review impact of climate change VBDs at Mediterranean on policy adaptation. No evaluation data was presented.
112	Ngindu, et. Al., 1990	Review on Water resources, not measure the intersectoral colaboration
113	Ngo, et al, 2009	A community based trial on Long lasting insecticide hammock for controlling malaria, but no intersectoal collaboration action was presented.
114	Nilsson, A., 2017	The role of International organization in making the norms. It's not related to VBDs
116	Nonaka, D., et al., 2009	Study on public and private health care provided malaria diagnosis and treatment. No intersectoral collaboration was measured.
117	Ohnishi, M. and K. Nakamura, 2009	Pre-and post-test of knowledge government and NGOs staff on environmental hygiene.
119	Okia, M., et al., 2016	Review malaria control program in Uganda for planning and implementation IVM.
120	Olveda, R., et al., 2010	Review Coordinating Research Neglected Parasitic Diseasein South East Asia

ID ref	Study (Name authors, year)	Reason of exclusion
121	Omumbo, J.A., et al., 2004	No collaboration with other sectors was presented
122	Owusu, N.O., 2014	Examine the levels of community participation in malaria control programmes
125	Ozbilgina, A., et al., 2011	Review the success of malaria control and strategies to achieve elimination in Turkey. No intersectoral collaboration was measured
126	Pao, M.L., 1992	Global and local collaborators for schistosomisis scientific collaboration.s.
127	Paquet, C, et.al., 1998	Control of infectious diseases in refugee and displaced populations in developing countries.
128	Patil, R.R., et al, 2011	Perception of elected representative in India on malaria. No evaluation data was presented.
129	Paul, C., et al., 2015	Literature review and perception of stakeholders on malaria policy. No evaluation data was presented.
131	Pronyk, P.M., et al., 2012	Review Millennium Development Goals and child survival in rural sub-Saharan Africa. Not related to VBD
132	Protopopoff, N., et al., 2007	Case study emergency response to malaria outbreak using IRS. No intersectoral collaboration action was presented.
134	Raulfs-Wang, C., et al., 2014	Meeting abstract on PEER supported by USAID in LMIC. No intersectoral collaboration is presented.
136	Ridley, R.G., 2004	Research on infectious diseases requires better coordination. No intersectoral collaboration was presented
137	Rojas, W., 2001	Community participation for integrated malaria control program. No intersectoral collaboration was presented
138	Roman, E., et al., 2014	Review malaria in pregnancy in three countries.
139	Rugemalila, J.B., et al., 2007	Report on multisectoral coordination on Malaria but not measure the intersectoral collaboration
140	Rutta, A.S.M., et al., 2012	Community participation for malaria early diagnosis and treatment and data collection. Not measure involving other sectors
141	Sambo, L.G., 2011	Perception of African leader for malaria control. No evaluation data and intersectoral collaboration action was presented.
142	Sanchez, L., et al., 2012	Testing semiparametric model in intersectoral collaboration. Similar data from original report in 2005 and 2009.
146	Savioli, L., et al., 2017	Opinion on Global Schistosomiasis, no intersectoral collaboration was measured
147	Schliessmann, D.J., et al., 1973	Vertical approach on malaria eradication program. No intersectoral collaboration was presented.

ID ref	Study (Name authors, year)	Reason of exclusion
148	Schwitz, F.M., et al., 2006	No ethical clearance. Discussed role of travel agent in general health information.
150	Sharma, S., et al., 2009	Report on Shifting public to private health care providers in India but not measure the intersectoral collaboration
151	Sharma, S., et al., 2014	Review VBDs program achievement and challenge. No intersectoral collaboration was measured
153	Sharp, B.L., et al.,2007	Malaria Control Collaboration, not intersectoral collaboration
154	Sibley, C.H., 2010	Review on monitring animalarial drug resistance, no intersectoral collaboration was measued
155	Siegel, E.R., 2001	Intersector collaboration on communication and connectivity for malaria program. No evaluation data was presented
156	Silva, R. and C.H. Paiva, 2015	Review on Malaria Elimination in Brazil, no intersectoral collaboration was measured
157	Singer, E., 2005	No intersectoral collabroration was measured
158	Sleigh, A., et al., 1998	Review Schistosomiasis eradication program in the prespective of political economy, management strategy and cost . Not measure intersectoral collaboration
159	Sleigh, A., et al., 1998	Review Schistosomiasis eradication program in the prespective of seetting, strategies, operations and outcomes.
160	Smith Gueye, C., et al., 2016	No evaluation/impact data was presented.
161	Sommerfeld, J.et al., 2012	A systematic review without additional new result study
162	Speelman, J. and G. van den Top, 1986	No intersectoral collaboration was measured, but there is statement that intersectoral collaboration between enginering and medical profesions is needed for longterm control of VBDs
163	Stone, C.M., et.al., 2014	A study on IVM against Malaria and LF but no intersectoral collaboration was measured
164	Suwannapong, N., et al.,2014	Cross sectional study on KAP community in dengue control. No intersectoral collaboration was measured.
165	Tambo, E., et al.,2016	Review on China-Africa Health Development Initiatives, systematic review
167	Tanner, M., 1994	Review a collaboration in developing health research capability in Tanzania.

ID ref	Study (Name authors, year)	Reason of exclusion
168	Taylor, E.M., et.al., 2014	Review on Politics and Anti-Politics of the GF experiments, not measure the intersectoral colaboration
169	The Lancet Infectious, D., 2010	Report on coordination essential for malaria batle, not measure the intersectoral collaboration
170	Tobgay, T., et al., 2013	Community - directed educational intervention for malaria elimination, no intersectoral collaboration involved
171	Toledo, M.E., et al., 2011	Short communication on the result of the research that was done in 2006.
172	Toledo M.E., et al.,2007	Community participation for dengue control. No intersectoral collaboration was measured.
173	Toledo Romani, M.E., et al., 2007	Community participation for dengue control
174	Tynan, et.al., 2011	Social and cultural aspect of treatment seeking behavior for Malaria in Vanuatu, no intersectoral collaboration was measured
175	Ubben, D. and E.M. Poll, 2013	Partnership to achieve international regulatory approval for use of Eurartesim, but no evaluated data was presented
177	Utzinger, J., et. 2013	A multidiciplinary alliance contributions for sustainable schistosomiasis control through innovation, validation and application of new tools and locally adapted intervention strategies complementary to preventive chemotherapy. No intersectoral collaboration was measured.
178	van den Berg, H. and W. Takken, 2006	Comment on Farmer Field School, not measure the intersectoral collaboration
179	van den Berg, H. and W. Takken, 2009	Integrated vector management not intersectoral collaboration
181	van den Berg, H., et al., 2007	A review based on finding and evaluation result
184	Wang C.H., et al, 2000	Community participation in dengue vector control. No other health sectors involvement.
186	Waren, et.al., 2013	GHI investment and health system strengthening, not measure intersectoral collaboration
187	Watanabe et al., 2015	Community participation in malaria elimination. No intersectoral collaboration was presented.
188	Wickramage, et.al., 2013	Case report on irregular migration as a potential source of malaria reintroduction in Sri Lanka, intersectoral collaboration is needed but not measured

ID ref	Study (Name authors, year)	Reason of exclusion
189	Wielgosz, B., et al., 2014	Correlations between agricultural and health outcome. NO intersectoral collaboration was presented.
190	Xu, J., et al., 2016	Review collaboration between China and Africa on schistosomiasis. No intersectoral collaboration was evaluated
192	Yewhalaw, D., et al, 2009	A study to assess the effect of man-made hydroelectric dam on malaria transmission and changing levels of prevalence in children, but not evaluate intersectoral collaboration
