# Supplementary material for: Intersectoral collaboration for the prevention and control of vector borne diseases to support the implementation of a global strategy: A systematic review
Source: PLoS One. 2018 Oct 10;13(10):e0204659. doi: 10.1371/journal.pone.0204659 (PMC6179246; doi:10.1371/journal.pone.0204659)
Supplement: S4 Table — (PDF) [file pone.0204659.s005.pdf]

S4 Table. Intervention, type of sector involved and results of the study

Authors, year	Vector Borne Disease addressed	Intervention/s	Type of sectors involved	Analysis method	Results	Indicators for intersectoral collaboration
Abeyewickreme, W., et al., 2012	Dengue	Education of dengue prevention and control, larva source management (distribution free of charge low cost compost bin, regular garbage collection, waste management at household level)	Policy makers, local government authorities, religious and local leaders, public health officials, NGOs, school principals and teachers; officers of the Central Environment Authority and other community leaders.	Student's t-tests (CI95%), a negative binomial model	No significant different of PPP between intervention and control group (p: 0.067) from baseline to final evaluation. Roles of stakeholders were identified.	Not measured
Afenyadu, G.Y., et al., 2005	Malaria	Treatment of presumtive malaria in school	Ghana Education Service, Primary school teachers	Proportion of corrective diagnosed and treatement compared between intervention and control area	75% of all malaria presumptive are treated correctly by teachers. The proportion of presumptive malaria correctly diagnosed and treated was significantly higher in the intervention area where pre-packaged chloroquine was used (P-value ¼ 0.00005, v2 ¼ 21).	Not measured
Arunachalam et al, 2012	Dengue	Education of dengue control, larval source management (containers cover, clean-up waste disposal)	School teachers and children, women group, community, NGOs, private sectors, health authorities.	Student t test (95% CI)	In intervention group: near 90% of respondents increased knowledge vector and disease transmission. In control group: knowledge was unchanged. There were significant different between intervention and control arm	Not measured

Authors, year	Vector Borne Disease addressed	Intervention/s	Type of sectors involved	Analysis method	Results	Indicators for intersectoral collaboration
					in HI (t:-14.7, p:0.012), CI (t:-6.06, p:0.0057), BI (t:-25.7, p:0.0011), PPI (t:0.35, p:0.0200)	
De Urioste-Stone, S.M., et al., 2015	Chagas diseases	Education of prevention and mechanical control of Chaga disease, a modified spraying method, organic waste management combined with productive household activities,	Health Sectors, Universities/research institutions, international donors, NGOs, community, health educators	Student's t test (CI95%) for KAP study. OR (95%CI) for intervention triatomine control, rodent control.	Significant different knowledge on biology of triatomine and Chagas disease after intervention (control vs intervention group, p<0.001). OR 10.6 (95%CI 3.2-34.8) persistent household-level re-infestation (pre-infestation vs post-infestation) in intervention area.	Not measured
					OR 8.3 (2.4–28.4, 95% CI) in infected early instars (control vs intervention)	
Deribew, A., et al., 2012	Malaria	Education of malaria prevention (trained 2105 head of households)	Focal malaria expert at the DHO, malaria committee at the DHO, the trained village residents, and the researchers.	Mean/proportion difference % (95% CI)<br><br>Crude and adjusted OR (95%CI)	Malaria prevalence was higher at the control villages compared to the intervention ones (proportion difference = -0.5; 95%CI: -2.5, 1.2). AOR = 0.73; 95%CI: 0.60, 0.89 of prevalence of Moderate anaemia in under-five children (control vs intervention).	Not measured
Johns, B., et al., 2016	Malaria	IRS using community-based and district-based		Chi-squared (95%CI)	No significant different of number of people protected	Not measured

Authors, year	Vector Borne Disease addressed	Intervention/s	Type of sectors involved	Analysis method	Results	Indicators for intersectoral collaboration
			Health sector, team of HEWs and team of local community	difference between groups in intervention, and t test (95%CI) for cost analysis.	in the DB IRS and CB IRS in 2013 and 2014 (P=0.09 for comparison between DB IRS and CB IRS).<br>No significant different of compliance Community-based (CB) IRS and DB (district-based) IRS districts in 2014.	
					Difference of cost per person protected in CB IRS compared to DB IRS US$0.16 in 2014 (US$0.86 vs. US $1.03, respectively; P=0.15)	
Kaatano, G.M., et al., 2015	Schistosomiasis and STH	Education using PHAST, constructed deep well, administering MDA by teacher	School teachers, head of households, research institute, NGO (GNI), international donor (JICA)	Non-parametric test for trend was performed to test for a trend of decreased prevalence of S. mansoni and hookworms with time (months/years).	In schoolchildren: declining trend prevalence of S. mansoni (z=-1.73, P=0.083), and for hookworms (z=-1.73, P=0.083. S. mansoni prevalence among schoolchildren was overall reduced by 90.5% and hookworms was reduced by 93.3% in 2013 compared to baseline.<br>Among adults, declining trend prevalence of S. mansoni (z=2, P=0.046), and hookworms (z=2, P=0.046). Overall reduction in the	Not measured

Authors, year	Vector Borne Disease addressed	Intervention/s	Type of sectors involved	Analysis method	Results	Indicators for intersectoral collaboration
					prevalence S. mansoni 83.2% (P= 0.0000), hookworms was 56.9% (P=0.0001).	
Kittayapong, P., et al., 2006	Dengue	Education prevention on dengue control, larva source management (garbage collection, a combination of locally produced Bti and the local copepod, local modified lethal ovitraps)	The heads of villages and schools, the Local Administrative Authority, the Local Public Health Office, and public health volunteers	Student t test (95% CI)	There was significant different between intervention vs control areas on larvae abundance (t= -3.297, df 150, P = 0.001) . DHF case rates declined from 265.25 to 0 at intervention village.	Not measured
Kittayapong, P., et al., 2012	Dengue	Community and stakeholders mobilization. Larva source management (screen net covers, mosquito traps portable vacuum aspirators at households. Using bio-control agent and biolarvicide)	Community leaders, local administrative authorities, municipal mayors, and local public health officer, ecohealth volunteers	Independent t -test (95%CI) to evaluate impact of vector densities. Paired t-test (95%CI) to compare the change of vector indices between intervention and control groups	The mean pupae per person index (PPI) was significantly different 0.19 (intervention) vs. 0.73 (control) (p=0.024) in the peak transmission season. There were no significant differences in HI, CI and BI indices between treatment and control groups at bimonthly surveyed interval	Not measured
Kittayapong, P., et al., 2008	Dengue	Clean-up campaign followed by weekly garbage pick-up, screen covers for water jars, a combination of copepod and Bti,	The Local Administrative Authorities, school children, householders, public health volunteers, and public health officers	Student t test (95% CI), and spatial analysis	There was significantly different average number of positive containers per house in the dengue foci and in the out of dengue foci (t= −3.493, P =0.001, df=150) in	Not measured

Authors, year	Vector Borne Disease addressed	Intervention/s	Type of sectors involved	Analysis method	Results	Indicators for intersectoral collaboration
		permethrin-treated lethal ovitraps			the intervention area. A reduction of the proportion of IgG–IgM positive students from13.46% to 0% in the intervention areas, while in control area shows an increase from 9.43% to 19.15%.	
Kusuma, Y.S., et al., 2017	Dengue	Health education message on dengue control	Municipal Corporation of Delhi (MCD), NGOs, construction companies, migrants' workers	Proportion different between pre and post intervention on awareness, knowledge, practices	A significant increase knowledge, awareness, practices in using personal protection and participation in awareness program post-intervention.	Not measured
Magnussen, P., et al., 2001	Schistosom iasis	Education on Schistomiasis control, prevention, diagnosis and treatment. Sanitation management of schools	District education office, district medical office, research team, headmaster of school, selected teachers, parents-teachers' associations, village health committees	Student's t-test (95% CI) and c2 test (95% CI).	A reduction of the overall prevalence of haematuria was 61.4% from 51.2% in 1995 to 20.1% in 1998.<br>A reduction of the geometric mean intensity of infection among positive by 60.6% from 71 eggs/l0 ml in 1995 to 28 eggs/l0 mL in 1996.	Not measured

Authors, year	Vector Borne Disease addressed	Intervention/s	Type of sectors involved	Analysis method	Results	Indicators for intersectoral collaboration
Okabayashi, et al. 2006	Malaria	Education of malaria control for school children	Health and education sectors (teacher, school principal, pupils)	McNemar test for change of knowledge and practice in teachers and schoolchildren	A significant increased skill the teachers to design a lesson plan on malaria (p = 0.015) and had taught about malaria p = 0.035). The schoolchildren significantly changed their behavior positively towards malaria prevention.	Not measured
Sanchez, 2009	Dengue	Education on dengue control, establish intersectoral committee at local level, community empowerment through community working group, community empowerment, routine dengue control program (entomological surveillance, source reduction, larviciding (temephos), adulticides with pyrethoids)	Heatlh council consisted of health sectors, community organizations, government authorities. Community working group (formal and informal leaders)	A two-way ANNOVAanova model using SAS procedure GLIMMIX	Areas with intersectoral coordination (CP4 and CP6) had significantly lower indices than the control area ($P < 0.05$). The area with the intersectoral coordination approach together with community empowerment maintained BI values below 0.1. These values continued to be significantly lower than the control area throughout the final evaluation period ($P = 0.004$).	Execution, decision making, evaluation

Authors, year	Vector Borne Disease addressed	Intervention/s	Type of sectors involved	Analysis method	Results	Indicators for intersectoral collaboration
Sanchez, 2005	Dengue	Education of dengue control (eliminating unused containers, covering tanks, cleaning public and inhibited areas), intersectoral group was trained and design social mobilization strategy and communication	Representatives of the government, community organizations, the Health Area, public services, Educational sector, Cultural sector, External experts from research institute	Differences in proportions before and after (95% CI)	In intervention area: knowledge and practice significantly improved, a reduction of HI from 3.72% to 0.61%. CI decreased from 0.275% to 0.05%. In control area: practice was not change, knowledge improved, HI fluctuated between 1.31% and 1.65%, CI increased from 0.1% to 0.3%.	Not measured
Sedlmayr, R., et al.,2013	Malaria	ITN distribution through private company	Private company supported in involved in identification and sensitization of the target population, storage, local distribution cost, distributed 40,000 ITNs and tracking utilization.	OR (95% CI) self-reported morbidity malaria. Proportion of ownership and utilization of ITNs	• Based on self-reported, farmers in the intervention has 42% lower odds of suffering a fever, and 49% lower odds of reporting a confirmed case of malaria.<br>• Coverage of ITNs utilization among children under five years in control group was 40% and in intervention group was 60%.<br>• Overall, mean increase in the likelihood of ITN utilization of approximately 15 % compared to MIS data 2010.	Not measured

Authors, year	Vector Borne Disease addressed	Intervention/s	Type of sectors involved	Analysis method	Results	Indicators for intersectoral collaboration
Tana S, et.al, 2012	Dengue	Community empowerment through local forum, intersectoral collaboration involvement, communication for school, community-hired entomology surveillance, the 3M campaign (clean, close and bury water containers)	Community members, political stakeholders, Community-based environmental health forums, Voluntary community workers, Women's association, health sector institutions, city office for environment affairs, public utility department, nongovernmental organizations, primary schools.	descriptive	Declining PPI from 0.015 to 0.005 in the intervention group, and from 0.05 to 0.04 in the control group. Declining Breteau index from 6.9 to 0.5 in the intervention group and increased from 2.0 to 3.5 in the control group. Increasing KAP in dengue prevention, community participation and its sustainability and ownership. Potential actors were identified	Not measured
Ulibarri, G., et al., 2016	Dengue, Chikungunya, Zika	Integrated approach for Aedes spp, through training of health workers, use of low-cost ecological ovillanta and community engagement.	Health workers and community, academic researchers, local health authorities from the Ministry of Health Vector Control Programme of Guatemala. international collaborators in Canada, Guatemala and Mexico	t-test for quantitative variable (egg count etc) and descriptive analysis for qualitative variable	• 80% of the student (health workers) were accredited (75-95 points of 100)<br>• The different of egg count between study site and control site was significant ($t=5.2577$; $p<0,05$), but within the study site households there is no statistically differences in the amount of Aedes eggs collected from ecological ovilantas and standard ovitraps	Not measured

Authors, year	Vector Borne Disease addressed	Intervention/s	Type of sectors involved	Analysis method	Results	Indicators for intersectoral collaboration
Vanlerberghe, V., et al., 2009	Dengue	Dengue control using community-based environmental management (established formal task force, intersectoral collaboration)	Community leaders, health sectors, private companies, government intersectoral groups	Time effect and group effect.	After intervention, house index, breteau index and pupae per inhabitant in intervention cluster lower than control cluster (HI: RR 0.49 (95%CI 0.27 - 0.88, p=0.018; BI: RR 0.48 (95%CI 0.26 - 0.88; PPI: RR 0.27 (95%CI 0.09 - 0.76).	Not measured
Wai, K.T., 2012	Dengue	Education of dengue control, vector control (chemical, biological, mechanical control), environmental management(waste-collection), establish ecohealth friendly group, intersectoral collaboration, local manufacture for produce low-cost lid cover	Ward authorities, midwives, members of Maternal and Child Welfare Association, key persons, school teachers, local manufacturers, volunteers	Proportion	The PPI decreased by 32% in intervention clusters by 54.5% reduction in control clusters in first evaluation.	Not measured
Yuan, L.P., et al., 2005	Schistosomiasis	Education of schistosomiasis for school children	Health and education sectors (teacher, school principal, pupils)	Chi-square test and multiple regression	A significant difference on knowledge, attitude and behaviour among intervention and control group post-intervention ($p<0.001$).	Not measured

Authors, year	Vector Borne Disease addressed	Intervention/s	Type of sectors involved	Analysis method	Results	Indicators for intersectoral collaboration
Argaw, M.D., et al.,2016	Malaria	Public Private partnership for malaria diagnosis, treatment, reporting to HMIS,	Profit and non-profif private health providers, USAID as donor for the project	Descriptive statical analysis, McNemar Chi square test for paired or dependent proportions.	Of 873,707 malaria suspected cases, 87.1 % was serviced in profit private facilities. Diagnosis confirmed by laboratory was improved from 87.7 % to 100.0 % in the last 3 months. Improved treatment adherence.	Not measured
Aumentado, C., et al., 2015	Dengue	Dengue treatment and vector control in emergency	Military, International and national donors and NGOs	Descriptive	A 61% increase in cases in 2014 compared to 2013 (n = 3254), but not exceeded the epidemic threshold. The Breteaux index decreased in many villages after intervention.	Not measured
Bhattacharya, S.K., Dash, A.P., 2017	Leishmaniasis	Diagnosis, treatment, vector control, surveillance, cross-border collarabotion among India, Bangladesh, Nepal	Department of Health India, Bangladesh, and Nepal, WHO and TDR, Indian Scientist, Pharmaceutical Company from German,	Descriptive	A reduction of Kala-Azar cases from 29,000 in 2010 to 8,243 in 2015 in India, as well as death cases-related to Kala-azar declined from 105 in 2010 to 5 in 2015.	Not measured
Castro, M.C., et al., 2009	Malaria	Education on drain cleaning, environmental management (drain cleaning) involved community, representative of city, municipal and ward	JICA, NMCP, Urban Malaria Control Programme (UMCP), community members, and city, municipal and ward representatives	A stepwise logistic regression (backward selection, p = 0.2)	There were significant reduction of malaria prevalance at EM area compared baseline after adjusted with age, rainfall, bed net use, and larviciding spray after April 2008(AOR 0.12, 95% CI	Not measured

Authors, year	Vector Borne Disease addressed	Intervention/s	Type of sectors involved	Analysis method	Results	Indicators for intersectoral collaboration
					0.05-0.3, p<0.001). Compared EM sites, control sites had a higher risk of infection after adjusted for age, rainfall, bed net use, and larviciding spray after March 2008 (AOR = 1.7, 95% CI 1.1–2.4, p = 0.0069).	
Chanda et al, 2008	malaria	Advocacy, legislation, social mobilization, IRS, ITN, environmental management, larva source management,	IVM working group consisted of public sectors (health, defense, environment, housing), research institutions, higher learning institutions, local authorities, private sectors and multi-/bilateral development partners.	Proportion and rate	An increase proportion of households with at least one ITN from 27% in 2001 to 53% in 2007. Malaria incidence rate declined from 424.0 in 2003 to 358.0 per 1000 population in 2007.	Not measured
Chandiwana, S.K., et al., 1991	Schistosomiasis	Snail control (a synthetic molluscicide), school screening and treatment, sanitation (the ventilated improved pit (VIP) latrine), water supplies (wells), health education,	District Development Fund (DDF), health sectors, education sector (teacher and students), the Blair Research Laboratory, community leaders.	Descriptive	A reduction prevalance of heavy infection of schistosomiasis in the targeted age group. Snail infection rates declined during the project implementation.	Not measured
Drameh, P.S.,et al., 2002	Onchocerciasis	Community mobilization, communication strategy, mass treatment administration	NGOs, Private company (Merck), WHO, World Bank, CDC, MoHs	Descriptive	An increased people treated by Ivermectin from 5.1 million per year in 1994 to over 24 million in 2000	Planning, communication, and regular reporting

Authors, year	Vector Borne Disease addressed	Intervention/s	Type of sectors involved	Analysis method	Results	Indicators for intersectoral collaboration
					through NGOs-assisted distribution channel.	
Gibbons, R.,V., et al., 2013	Dengue, Japanese Ensephalitis	Research on diagnosis, treatment, and vaccine development of Dengue and Japanese Ensephalitis	Kamphaeng Phet Province Authorities, Ministry of Public Health (MoPH) Thailand, US army and navy medical research, Japan International Cooperation Agency (JICA), the Deputy Prime Minister of Thailand, members of Parliament, the Ministers of Health and Education, and the provincial governor, village heads, directors of primary and secondary schools, teachers, and parents	Descriptive	JE vaccine developed and included in EPI program, reduction of Japanese Ensephalitis cases reported.	Not measured
Ghosh, S.K., et al., 2006	Malaria	Education on malaria control using traditional theater	Inter-sectoral committee consisted of 10 government and non-government organizations, research institution, Departments of Health, Education, Child and Women's	Fisher Exact and $\chi 2$ tests exposed and non-exposed groups	Significant difference in an increase of knowledge on malaria between exposed and non-exposed group ($p<0,001$)	Not measured

Authors, year	Vector Borne Disease addressed	Intervention/s	Type of sectors involved	Analysis method	Results	Indicators for intersectoral collaboration
			Welfare, Rural Development and Panchayat Raj, Tumkur Science Forum, local political and religious leaders, political leaders			
Herdiana, et al., 2013	Malaria	Malaria control program, ITNs distribution, diagnosis and treatment, surveillance and reporting system	NMCP, District Health Office (DHO), District Planning Board, Civil Registration Office, District Health Office, Health Division of the Armed Forces, Center of Data and Information under the Mayor of Sabang, and sub district leaders, WHO, UNICEF, Eijkman Institute, Gadjah Mada University, NGOs (Mentor Initiative)	Descriptive	A nearly 30-fold decline in malaria incidence from 3.83 to 0.13 per thousand population from 2008 to 2011	Not measured
Ho, L.L., et al., 2017	Zika	Education on Zika, dengue and chikungunya, vector control in the aircfart	Health port office and private sectors (tour guides, aircraft companies)	Proportion confirmed case of dengue, chikungunya, and zika	Of 13 Zika imported cases, five cases found at airport screening.	Not measured

Authors, year	Vector Borne Disease addressed	Intervention/s	Type of sectors involved	Analysis method	Results	Indicators for intersectoral collaboration
Ichimori, K., and Crump., A., 2005	Lymphatic Filariasis	MDA, vector control	22 member countries authorities, the government of Australia, the government of Japan, WHO, pharmaceutical companies (GSK, Merck)	Descriptive	Adopted strategy of MDA to all 22 member countries.	Not measured
Kong, X., et al.,2017	Malaria	Malaria surveillance, diagnosis, treatment	CDC of China, Entry-Exit Inspection and Quarantine Bureau,<br>Public Security Bureau, and Education Bureau	Descriptive	Malaria indigenous case declinded from 52 cases in 2005 to null between 2012 and 2015.	Not measured
Krisher, L.K., et al., 2016	Malaria	Surveillance and response, operational research, treatment, vector control in cross-border	Malaria control program from Peru and Ecuador, WHO/PAHO, CDC US, USAID, private health providers.	Descriptive	A significant reduction of malaria incidence in both countries.	Not measured
Martins, J.S. et al., 2012	Malaria	Provided financial support for malaria control program	Health sectors, research institutions, international donors, NGOs, private sectors	Proportion of annual malaria incidence (AMI). Content and thematic analyses, and triangulation qualitative and quantitative data	A reduction of the AMI reduction was around 10.1 % (95%CI 9.6 – 11) P<0.001 in 2007 compared to baseline in 2004. GFATM contributed to health system (surveillance, diagnosis, vector control management). • Health education interventions improved malaria knowledge of community.	Not measured

Authors, year	Vector Borne Disease addressed	Intervention/s	Type of sectors involved	Analysis method	Results	Indicators for intersectoral collaboration
Murhandarwati, E.E., et al., 2015	Malaria	Community-based active surveillance system	A village health forum (Forum Kesehatan Desa) and Vigilant Village Programme (Desa Siaga), the Department of Transportation, bus agencies, local authorities	Descriptive	An increase number of malaria cases by 1.5-fold in 2011 compared to data 2007. Deduction of budget allocation caused bounce back of malaria cases after eliminated.	Not measured
Mutero, C.M., et al., 2015	Malaria	Implementation of Integrated Vector Management	Health department, research institution (KEMRI and ICIPE), other sectors (Municipal Council, ministries of fisheries, environment, and natural resources), community-based groups, Biovision Foundation.	Chi-square test, a zero-inflated negative binomial (ZINB) model, a zero-inflated Poisson (ZIP) model	A reduction of proportion malaria cases among children admitted in Malindi Hospital from 23.7% in 2006 to 10.47% in 2011 ($p < 0.001$). While in Nyabondo, the proportion was increased from 24.5% in 2009 to 30.3% in 2011.<br>In Malindi, a significant decline in the density of An. gambiae and other vectors over the years ($p = 0.001$), and a significant association between vector abundance and year. In Nyabondo, an increase of vectors densities over years.	Not measured
Njau, R.J., et al., 2009	Malaria	Distribution of ITNs using subsidy scheme	Medical Officer, NMCP, ITN Cell, multi/bilateral international agencies (the UK Department for International	Chi square tests (95% CI)	There was significant increase proportion of household owning mosquito nets in second survey (56.05%) compared to	Not measured

Authors, year	Vector Borne Disease addressed	Intervention/s	Type of sectors involved	Analysis method	Results	Indicators for intersectoral collaboration
			Development, the Swiss Development Corporation, PMI, the Irish AID, WHO, UNICEF), NGOs, private sectors, research institutes, FBOs.		baseline (41.8%) (p < 0.001).<br><br>There was a significant increase in mosquito net ownership in the most-poor quintile, followed by the second quintile.	
Owusu, N.O., et al., 2013	Malaria	Malaria control program	health sector, agriculture, education, environment and economic/finance sectors, service providers, administrators, service users/community members, local politicians	A two-level multinomial multilevel ordered logistic regression technique.	The overall level of integration at Ahafo Ano South district was 2.9 and 2.4 for the Kumasi Metropolis (p<0.05).	The type of institution, level of focus on malaria and source of funding, district effect (rural vs urban)
Oyediran, et al, 2002	Malaria	Treatment	Private company (Glaxo Wellcome), MoH Uganda, MoH Kenya, WHO, DFID, NGOs	Descriptive	Of the clinical cases, 1101 (0.68%) received directly observed treatment with Malarone. Patient compliance with follow up exceeded 80%.	Not measured
Peters, D.H., and Phillips, T., 2004	Onchocerciasis	Mass distribution of drug	NGOs, Private company (Merck), WHO, World Bank, CDC, MoHs	Descriptive	Strong leadership from senior leaders, common agreement of priorities,	Governance and management factors

Authors, year	Vector Borne Disease addressed	Intervention/s	Type of sectors involved	Analysis method	Results	Indicators for intersectoral collaboration
Qunhua, L., et al., 2003	Malaria	Environmental management (new farming method)	Farmers, central government, international donors, agriculture and health sectors	Proportion, mean and incidence rate per 10,000 population	A significant reduction of malaria incidence and population of adult mosquito at area that applied wet/dry crop rotation.	Not measured
Renggli, S., et al., 2013	Malaria	Distribution of ITNs, social mobilization, monitoring and evaluation	Red Cross, community, local government authorities (WEO = Ward Executive Officer; VEO/MEO = Village/Street Executive Officer), mass media, NCMP, NGOs,	Proportion of bed nets ownership, cost analysis	Coverage ownership of nets reached 91.5% in mainland Tanzania.<br>A financial cost per LLIN delivered of USD 5.30 (included production, transport and campaign)	Not measured
Sanders et al, 2014	Malaria	Diagnosis, treatment, surveillance, IRS, ITNs distribution	Malaria control program, plantatation companies, workers	Descriptive	A decline malaria incidence from 49,192 cases in 1994 to 2,032 cases in 2011 in Sabah state.	Not measured
Sharp B., et al., 2002	Malaria	IRS for malaria control, public / private sector collaboration	Spray operators were recruited from the community and trained Representatives from the Zambian Environmental Council audited as independent observer	Student's two-sample t-test	Reduction of malaria incidence rate ratio of 0.65 (95% CI 0.44, 0.97).<br><br>The overall unit cost of the residual spraying programme is cheaper than providing insecticide-treated bed nets	Not measured

**Authors, year**	**Vector Borne Disease addressed**	**Intervention/s**	**Type of sectors involved**	**Analysis method**	**Results**	**Indicators for intersectoral collaboration**
van den Berg, H., et al., 2012	Malaria and dengue	Malaria interventions based on micro-stratification (prevention (bed nets, IRS), diagnosis type), community participation,	Government officers, barangay health workers, members of civil society, community, school, taxi association, education department, mining sector, barangay chiefs and councillors	Descriptive	A reduction of malaria cases in two provinces, two villages.	Not measured
Wangroongsarb, Y., et al.,1997	Dengue	Education of dengue control, larva source management (larvaciding)	Ministry of public health, Ministry of education at central, province and district level ad also Division Environmental Health, Municipality at province and district level	Proportion	A reduction of Dengue morbidity rate among schoolchildren and other population occurred in 62.5% of the provinces and 64.3% of the districts surveyed.<br>76.7% of the schools trained the project, 80% of the teachers taught larval control activities, 94.4% of students involved in the activities.	Not measured
Xu, J.W., et al., 2016	Malaria	Diagnosis, treatment, communication, ITN distribution, migrant malaria workers (MMW), village malaria workers (VMW)	MoH China, MoH Myanmar, GFATM, NGO (HPA),	Risk ratio (RR) for prevalance and incidence. Thematic method for FDG and indepth-interview	In Myanmar, an 89 % reduction of Prevalance parasite rate (RR: 0.11, 95 % CI, 0.09–0.14). In China, Compared with the API in 2008, API rate ratio (IRR) was only 0.17 (95 % CI, 0.16–0. 18) in 2013.	Not measured
Zhang, J., et al, 2016	Malaria	Malaria control program (education, diagnosis,	NGO (HPA), local government, health sector,	Proportion and rate	Malaria parasite rate (MPR) in the five Myanmar areas has declined from 13.63 %in	Not measured

Authors, year	Vector Borne Disease addressed	Intervention/s	Type of sectors involved	Analysis method	Results	Indicators for intersectoral collaboration
		treatment, surveillance, distribution LLINs)	International donor (GFATM)		2008 to 1.50 % in 2013. No malaria deaths were reported in 2013. An increased proportion of household using LLINs from 28.3% in 2008 to 57.3% in 2013.	
