# Supplementary material for: Intersectoral collaboration for the prevention and control of vector borne diseases to support the implementation of a global strategy: A systematic review
Source: PLoS One. 2018 Oct 10;13(10):e0204659. doi: 10.1371/journal.pone.0204659 (PMC6179246; doi:10.1371/journal.pone.0204659)
Supplement: S5 Table — (PDF) [file pone.0204659.s006.pdf]

S5 Table. Pre and Post Intervention from intervention studies (n=21)

Intervention	Study ID	Study design	Prevalence or incidence				Percent reduction	Outcome Measured
			Pre-intervention		Post-intervention			
			Control	Intervention	Control	Intervention		
Education of dengue prevention and control. larva source management (distribution free of charge low cost compost bin. regular garbage collection. waste management at household level), involved volunteer and schoolchildren	Abeyewickreme. W. et al. 2012	RCT	3.40	4.00	1.00	0.10	97.5%	Pupae per 100 persons Index
Education of dengue control through women group and schoolchildren, larval source management (containers cover, clean-up waste disposal)	Arunachalam et al. 2012	RCT	0.729	1.075	0.355	0.004	99.6%	Pupae per person index
			17.2	19.6	16.5	4.2	78.6%	House index (%)
			7.51	8.91	5.72	1.05	88.2%	Container Index (%)
			22.2	30.8	21.4	4.3	86.0%	Breteau Index (%)
Community and stakeholders' mobilization, establish eco-health volunteer. Larva source management (screen net covers, mosquito traps portable vacuum aspirators at households. Using bio-control agent and biolarvicide)	Kittayapong. P. et al. 2012	RCT	0.42	0.44	0.4	0.04	90.9%	Pupae per person index
			38.84	37.19	14.03	11.68	68.6%	House Index
			11.19	9.20	5.38	3.01	67.3%	Container Index
			78.79	81.86	21.49	24.46	70.1%	Breteau Index
Education on dengue control, establish intersectoral committee at local level, community empowerment through community working group, routine dengue control program (entomological surveillance, source reduction, larviciding (temephos), adulticides with pyrethoids)	Sanchez. 2009	Controlled before-after (CBA)	0.30	1.05	0.20	0.05	95%	Bretaeu Index (BI)
Education of dengue control (eliminating unused containers, covering tanks, cleaning public and inhibited areas), intersectoral group was trained and design social mobilization strategy and communication	Sanchez. 2005	Controlled before-after (CBA)	1.31	3.72	1.65	0.61	84%	House index (%)
			0.1	0.27	0.3	0.06	77.8%	Container Index (%)
Community empowerment through local forum, intersectoral collaboration involvement, communication for school, community-hired entomology surveillance, the 3M campaign (clean, close and bury water containers)	Tana S. et.al. 2012	RCT	0.05	0.015	0.04	0.005	66.7%	Pupae per person index
			2.0	6.9	3.5	0.5	92.8%	Breteau index

Intervention	Study ID	Study design	Prevalence or incidence				Percent reduction	Outcome Measured
			Pre-intervention		Post-intervention			
			Control	Intervention	Control	Intervention		
Larcaviding with temephos. selective adulticiding with cyoermethrin or clorpyriphos. communication and education. surveillance entomology. established formal task force/community working group. formulation local steering committee consisted relevant stakeholders	Vanlerberghe. V. et al. 2009	RCT	0.2	0.25	0.48	0.26	- 4%	House index (%)
			0.2	0.27	0.52	0.28	-3.7%	Breteau Index (per 100 houses)
			0.29	0.44	1.40	0.36	18.2%	Pupae per inhabitant ($*10^{-3}$)
Education of dengue control, vector control (chemical, biological, mechanical control), environmental management(waste-collection), establish ecohealth friendly group, intersectoral collaboration, local manufacture for produce low-cost lid cover	Wai. K.T. 2012	RCT	0.51	0.48	0.15	0.2	58.3%	Mean pupae per person index
Treatment of presumtive malaria in school by teacher	Afenyadu. G.Y. et al. 2005	RCT	NA	NA	88.7%	78.9%	NA	Correct presumptive diagnosis of malaria
			NA	NA	86.6%	97%	NA	Proportion of correctly diagnosed presumptive malaria cases adequately treated
Education of prevention and mechanical control of Chagas disease, a modified spraying method, organic waste management combined with productive household activities,	De Urioste-Stone. S.M. et al. 2015	RCT	19	19.4	5.9	7.9	59.3%	Domiciliary triatomine infestation (%)
Education of malaria prevention (trained 2105 head of households)	Deribew. A. et al. 2012	RCT	8.3	10.5	6.7	6.2	41.0%	Prevalence (%) of malaria
IRS using community-based and district-based	Johns. B.. et al.. 2016	RCT	99.60	99.30	0.20	99.20	0.1%	Spray coverage rate
Education using PHAST, constructed deep well, administering MDA by teacher	Kaatano. G.M. et al. 2015	Interrupted time series (ITS)	NA	42.5	NA	4.0	90.6%	Prevalence *S.mansoni* in schoolchildren
			NA	31.7	NA	5.3	83.3%	Prevalence *S.mansoni* in adult
Education prevention on dengue control through local leaders, larva source management (garbage collection, screen	Kittayapong. P. et al. 2006	Controlled before-after (CBA)	217.86	265.25	322.23	0	100%	DHF case rate per 100,000 population

Intervention	Study ID	Study design	Prevalence or incidence				Percent reduction	Outcome Measured
			Pre-intervention		Post-intervention			
			Control	Intervention	Control	Intervention		
cover, a combination of locally produced Bti and the local copepod, local modified lethal ovitraps)								
Clean-up campaign followed by weekly garbage pick-up by local administrative, screen covers for water jars, a combination of copepod and Bti, permethrin-treated lethal ovitraps	Kittayapong. P. et al. 2008	RCT	9.4	13.5	19.2	0.0	100%	% IgG-IgM positive of dengue case
			35	38	49	1	97.4%	% Positive container
Education on Schistomiasis control, prevention, diagnosis and treatment. Sanitation management of schools	Magnussen. P. et al. 2001	Time series design	NA	51.2	NA	23.4	54.3%	Prevalence haematuria among schoolchildren
Education of malaria control for primary school children (teaching aids, teacher training)	Okabayashi. et al. 2006	Uncontrolled before-after	NA	25.0	NA	4.8%	80.8%	Proportion children never heard about malaria (%)
			NA	30.7	NA	47.7	-55.4%	Proportion teacher can design teaching plan of malaria (%)
ITN distibution through private company	Sedlmayr. R. et al..2013	RCT	34	34	25	40	-15%	Proportion utilization of ITN at children under five
			23	23	27	15	34.8%	Proportion self-reported fever at children under five
Integrated approach for Aedes spp, through training of health workers, use of low-cost ecological ovillanta and community engagement.	Ulibarri. G. et al. 2016	RCT	2296	19073	4875	34820	79.9%	Monthly total eggs destroyed
Education of schistosomiasis for school children	Yuan. L.P. et al. 2005	Controlled before after	NA	NA	86.3	99.3	NA	% children had heard about schistosomiasis
