# Supplementary material for: Intersectoral collaboration for the prevention and control of vector borne diseases to support the implementation of a global strategy: A systematic review
Source: PLoS One. 2018 Oct 10;13(10):e0204659. doi: 10.1371/journal.pone.0204659 (PMC6179246; doi:10.1371/journal.pone.0204659)
Supplement: S6 Table — (PDF) [file pone.0204659.s007.pdf]

**S6 Table. Roles of multi-sectoral organization in VBDs control and prevention**

| Type of sector | Part of sector involved | Access | Technical support | Financial support | Social mobilization | Policy development | Planning | Logistics | Implement the activities | Monitoring & Supervision |
|---|---|---|---|---|---|---|---|---|---|---|
| **Education** | **Department** | [1-8] | [7, 9, 10] | [7] | [10, 11] | [1] | [4, 9, 11, 12] | | | [3] |
| | **Teacher** | | [3, 8] | | [2, 3, 5-8, 13-16] | | [5] | | [1, 4-6, 8, 14, 16, 17] | |
| | **Students** | | | | [2, 13, 15, 16] | | | | [4-7, 16] | |
| **Agriculture** | **Department** | [4] | [18, 19] | [4, 18] | [18] | [18] | [18] | [19] | [4, 18] | |
| **Military** | **Department** | | [20-22] | [21] | | [21] | [9, 20-22] | [20] | | [21] |
| | **Army** | | | | | | | | [20, 22] | |
| **Health** | **Department** | [20, 21, 23-25] | [4, 6, 7, 9-12, 15, 19, 20, 22-37] | [4, 7, 22, 26, 32, 38] | [11, 13, 20, 36] | [4, 9, 21, 23-25, 28, 29, 32, 37, 39] | [7-12, 16, 19, 22, 23, 26-29, 31-35, 37-42] | [3, 4, 7, 15, 22, 27, 28, 34, 41, 42] | [29] | [3-5, 7, 8, 10-12, 22-26, 28, 30, 32-35, 37, 39, 40, 43] |
| | **Health workers (doctor, nurse, midwives, epidemiologist, entomologist, health promotor, etc)** | | [2, 3, 13, 16, 44] | [2] | [10, 13, 19, 22, 24, 31, 38, 44] | [2] | [2, 44] | | [8, 12, 14, 16, 20, 22-25, 28, 30-32, 34, 37, 39, 45] | [16, 17, 33, 44, 46, 47] |
| **Immigration** | **Department** | [12, 22] | | | | | [12, 26] | | [12] | |
| **Public security** | **Department** | [12] | | | | | [12] | | [12] | |
| **Cultural** | **Department** | | [11] | | [11] | | [11, 15] | | | |
| **Labour** | **Department** | [26] | | | [26] | | [26] | | | |
| **Public work** | **Department** | [11] | [2, 11] | [11] | [11] | | [11] | | | |
| **Fishery** | **Department** | [4] | [4] | | | | | | [4] | |
| **Housing** | **Department** | | [9] | | | | [9] | | | |

| Type of sector | Part of sector involved | Access | Technical support | Financial support | Social mobilization | Policy development | Planning | Logistics | Implement the activities | Monitoring & Supervision |
|---|---|---|---|---|---|---|---|---|---|---|
| **Environment** | **Department** | | [9] | 123 | | | [9] 123 | | 123 | |
| **Tourism** | **Department** | | [4] | | [4] | | | | [4] | |
| **Rural development** | **Department** | | | | | | [15] | | | |
| **Civil registration office/ center of database** | **Department** | [22] | | | | [22] | [22] | | | |
| **Child and women welfare** | **Department** | | | | | | [15] | | | |
| **Central/Local authorities** | | [2, 4, 8, 11, 14, 21, 22, 41, 42, 48] | [9, 11, 18, 33] | [11, 13, 14, 22, 28, 33, 38, 42, 48] | [9, 11, 38] | [4, 11, 13, 18, 22, 28, 48] | [6, 9, 11, 12, 14, 18, 22, 28, 33, 38, 41, 42, 48] | [13, 14, 41, 42] | [6, 11, 14] | [11, 14, 18, 33] |
| **NGOs** | | | [17, 19, 23, 25, 37, 46, 48] | [22, 25, 29, 31, 49] | [9, 17, 25, 31, 32, 37, 48, 49] | | [9, 17, 23, 25, 29, 31, 37, 48, 49] | [17, 20, 22, 23, 25, 29, 37, 47, 48] | [9, 15, 17, 20, 22, 23, 25, 29, 31, 32, 37, 46, 48, 49] | [23, 25, 32, 46, 49] |
| **Private company** | **Bank** | | | [2] | | | | | | |
| | **Pharmacy company** | | | [30, 31, 39, 45, 49] | | | | [30, 31, 39, 45, 49] | | |
| | **Plantation/ agriculture** | [43] | | [34, 43] | [34, 43] | | [34, 43] | [34] | [34, 43] | [43] |
| | **Mining** | [35] | | [35] | [35] | | [35] | [35] | [35] | [35] |
| | **Waste management** | [2] | | | | | | | | |
| | **Insecticide industry** | | | | | | | [19] | | |

| **Type of sector** | **Part of sector involved** | **Access** | **Technical support** | **Financial support** | **Social mobilization** | **Policy development** | **Planning** | **Logistics** | **Implement the activities** | **Monitoring & Supervision** |
|---|---|---|---|---|---|---|---|---|---|---|
| | **Local manufacture** | [46] | [44] | [46] | [46] | | [46] | [46] | [10, 44, 46] | [44, 46] |
| | **Tourism/ tranportation industry** | [28] | | | [6, 26, 28] | | | | [6, 26] | |
| **International donor/agencies** | **i.e USAID, GFATM, DFID, JICA, PMI, RBM, WHO, UNICEF, etc** | | [9, 20, 22, 24, 25, 29-31, 38, 39, 45, 49] | [17, 19, 20, 22-25, 27, 29-31, 37-39, 45, 48, 49] | | [22, 39, 45] | [20, 22, 24, 25, 31, 39, 45, 49] | [22, 24, 27, 30, 43] | | [20, 22, 24, 30, 31, 45, 49] |
| **Red Cross** | | | | | [32] | | | | | |
| **CBO** | **Women group** | | | | [2, 39, 47] | | | | [39] | |
| **Community** | **Community leaders** | | | | [10, 19, 22, 41, 42] | | [22, 41, 42] | | [10] | |
| | **Volunteers/ householders** | | | | [3, 6, 11, 13, 16, 22, 24, 28, 32, 35, 36, 38, 42, 46, 47] | | [11] | | [3, 6, 14, 16, 17, 22, 24, 27, 28, 32, 35-37, 40-42] | [41, 42] |
| | **Religious leaders** | | | | [13, 15] | | | | [15] | |
| | **Farmers/ plantation labour** | | | | [18] | | | | [18, 34, 43] | |
| | **Forum** | | | | [10, 11, 28, 33] | [28] | [28, 33, 40, 44, 47] | | [10, 11, 33, 40] | [33, 40] |
| **External expert** | **University** | | [9, 16, 19, 21, 36, 42] | [42] | [11] | | [8, 9, 11] | [42] | | [8, 16, 42] |

| Type of sector | Part of sector involved | Access | Technical support | Financial support | Social mobilization | Policy development | Planning | Logistics | Implement the activities | Monitoring & Supervision |
|---|---|---|---|---|---|---|---|---|---|---|
| | **Research institution** | | [4, 9-11, 14-16, 21, 39, 40, 45, 46] | [11] | | | [4, 5, 9-11, 14, 15, 21, 39, 40, 45, 46] | | | [5, 10, 14, 16, 21, 30, 40, 45, 46] |
| **Media** | | | | | [4, 15, 32, 38] | | | | | |
| **Political leader** | | | | | | [15, 29, 47] | [15, 29, 47] | | | |

**References**


1. Afenyadu GY, Agyepong IA, Barnish G, Adjei S: **Improving access to early treatment of malaria: a trial with primary school teachers as care providers.** *Trop Med Int Health* 2005, **10:**1065-1072.
2. Arunachalam N, Tyagi BK, Samuel M, Krishnamoorthi R, Manavalan R, Tewari SC, Ashokkumar V, Kroeger A, Sommerfeld J, Petzold M: **Community-based control of Aedes aegypti by adoption of eco-health methods in Chennai City, India.** *Pathog Glob Health* 2012, **106:**488-496.
3. Magnussen P, Ndawi B, Sheshe AK, Byskov J, Mbwana K, Christensen NØ: **The impact of a school health programme on the prevalence and morbidity of urinary schistosomiasis in Mwera Division, Pangani District, Tanzania.** *Transactions of the Royal Society of Tropical Medicine and Hygiene* 2001, **95:**58-64.
4. Mutero CM, Mbogo C, Mwangangi J, Imbahale S, Kibe L, Orindi B, Girma M, Njui A, Lwande W, Affognon H, et al: **An Assessment of Participatory Integrated Vector Management for Malaria Control in Kenya.** *Environ Health Perspect* 2015, **123:**1145-1151.
5. Okabayashi H, Thongthien P, Singhasvanon P, Waikagul J, Looareesuwan S, Jimba M, Kano S, Kojima S, Takeuchi T, Kobayashi J, Tateno S: **Keys to success for a school-based malaria control program in primary schools in Thailand.** *Parasitology International* 2006, **55:**121-126.
6. van den Berg H, Velayudhan R, Ebol A, Catbagan BH, Jr., Turingan R, Tuso M, Hii J: **Operational efficiency and sustainability of vector control of malaria and dengue: descriptive case studies from the Philippines.** *Malar J* 2012, **11:**269.
7. Wangroongsarb Y: **Dengue Control through Schoolchildren in Thailand.** *Dengue Bulletin* 1997, **21:**52-62.
8. Yuan LP, Manderson L, Ren MY, Li GP, Yu DB, Fang JC: **School-based interventions to enhance knowledge and improve case management of schistosomiasis: a case study from Hunan, China.** *Acta Trop* 2005, **96:**248-254.
9. Chanda E, Masaninga F, Coleman M, Sikaala C, Katebe C, Macdonald M, Baboo KS, Govere J, Manga L: **Integrated vector management: the Zambian experience.** *Malar J* 2008, **7:**164.
10. Vanlerberghe V, Toledo ME, Rodriguez M, Gomez D, Baly A, Benitez JR, Van der Stuyft P: **Community involvement in dengue vector control: cluster randomised trial.** *Bmj* 2009, **338:**b1959.
11. Sanchez L, Perez D, Perez T, Sosa T, Cruz G, Kouri G, Boelaert M, Van der Stuyft P: **Intersectoral coordination in Aedes aegypti control. A pilot project in Havana City, Cuba.** *Trop Med Int Health* 2005, **10:**82-91.

12. Kong XL, Liu X, Tu H, Xu Y, Niu JB, Wang YB, Zhao CL, Kou JX, Feng J: **Malaria control and prevention towards elimination: data from an eleven-year surveillance in Shandong Province, China.** *Malaria Journal* 2017, **16**.
13. Abeyewickreme W, Wickremasinghe AR, Karunatilake K, Sommerfeld J, Axel K: **Community mobilization and household level waste management for dengue vector control in Gampaha district of Sri Lanka; an intervention study.** *Pathog Glob Health* 2012, **106:**479-487.
14. Chandiwana SK, Taylor P, Matanhire D: **Community control of schistosomiasis in Zimbabwe.** *Central African journal of medicine,* **37:**69.
15. Ghosh SK, Patil RR, Tiwari S, Dash AP: **A community-based health education programme for bio-environmental control of malaria through folk theatre (Kalajatha) in rural India.** *Malar J* 2006, **5:**123.
16. Kittayapong P, Yoksan S, Chansang U, Chansang C, Bhumiratana A: **Suppression of dengue transmission by application of integrated vector control strategies at sero-positive GIS-based foci.** *Am J Trop Med Hyg* 2008, **78:**70-76.
17. Kaatano GM, Siza JE, Mwanga JR, Min DY, Yong TS, Chai JY, Ko Y, Chang SY, Kullaya CM, Rim HJ, et al: **Integrated Schistosomiasis and Soil-Transmitted Helminthiasis Control over Five Years on Kome Island, Tanzania.** *Korean J Parasitol* 2015, **53:**535-543.
18. Qunhua L, Xin K, Changzhi C, Shengzheng F, Yan L, Rongzhi H, Zhihua Z, Gibson G, Wenmin K: **New irrigation methods sustain malaria control in Sichuan Province, China.** *Acta Trop* 2004, **89:**241-247.
19. De Urioste-Stone SM, Pennington PM, Pellecer E, Aguilar TM, Samayoa G, Perdomo HD, Enriquez H, Juarez JG: **Development of a community-based intervention for the control of Chagas disease based on peridomestic animal management: an eco-bio-social perspective.** *Trans R Soc Trop Med Hyg* 2015, **109:**159-167.
20. Aumentado C, Cerro BR, Olobia L, Suy LL, Reyes A, Kusumawathie PH, Sagrado M, Hall JL, Abeyasinghe R, Foxwell AR, Vestergaard LS: **The prevention and control of dengue after Typhoon Haiyan.** *Western Pac Surveill Response J* 2015, **6 Suppl 1:**60-65.
21. Gibbons RV, Nisalak A, Yoon IK, Tannitisupawong D, Rungsimunpaiboon K, Vaughn DW, Endy TP, Innis BL, Burke DS, Mammen MP, Jr., et al: **A model international partnership for community-based research on vaccine-preventable diseases: the Kamphaeng Phet-AFRIMS Virology Research Unit (KAVRU).** *Vaccine* 2013, **31:**4487-4500.
22. Herdiana H, Fuad A, Asih PB, Zubaedah S, Arisanti RR, Syafruddin D, Kusnanto H, Sumiwi ME, Yuniarti T, Imran A, et al: **Progress towards malaria elimination in Sabang Municipality, Aceh, Indonesia.** *Malar J* 2013, **12:**42.
23. Argaw MD, Woldegiorgis AG, Abate DT, Abebe ME: **Improved malaria case management in formal private sector through public private partnership in Ethiopia: retrospective descriptive study.** *Malar J* 2016, **15:**352.
24. Krisher LK, Krisher J, Ambuludi M, Arichabala A, Beltran-Ayala E, Navarrete P, Ordonez T, Polhemus ME, Quintana F, Rochford R, et al: **Successful malaria elimination in the Ecuador-Peru border region: epidemiology and lessons learned.** *Malar J* 2016, **15:**573.
25. Martins JS, Zwi AB, Kelly PM: **Did the first Global Fund grant (2003-2006) contribute to malaria control and health system strengthening in Timor-Leste?** *Malar J* 2012, **11:**237.
26. Ho LL, Tsai YH, Lee WP, Liao ST, Wu LG, Wu YC: **Taiwan's Travel and Border Health Measures in Response to Zika.** *Health Secur* 2017, **15:**185-191.
27. Johns B, Yihdego YY, Kolyada L, Dengela D, Chibsa S, Dissanayake G, George K, Taffese HS, Lucas B: **Indoor Residual Spraying Delivery Models to Prevent Malaria: Comparison of Community- and District-Based Approaches in Ethiopia.** *Glob Health Sci Pract* 2016, **4:**529-541.
28. Murhandarwati EE, Fuad A, Sulistyawati, Wijayanti MA, Bia MB, Widartono BS, Kuswantoro, Lobo NF, Supargiyono, Hawley WA: **Change of strategy is required for malaria elimination: a case study in Purworejo District, Central Java Province, Indonesia.** *Malar J* 2015, **14:**318.
29. Njau RJ, de Savigny D, Gilson L, Mwageni E, Mosha FW: **Implementation of an insecticide-treated net subsidy scheme under a public-private partnership for malaria control in Tanzania--challenges in implementation.** *Malar J* 2009, **8:**201.

30. Oyediran AB, Ddumba EM, Ochola SA, Lucas AO, Koporc K, Dowdle WR: **A public-private partnership for malaria control: lessons from the Malarone Donation Programme.** *Bull World Health Organ* 2002, **80:**817-821.
31. Peters DH, Phillips T: **Mectizan Donation Program: evaluation of a public-private partnership.** *Trop Med Int Health* 2004, **9:**A4-15.
32. Renggli S, Mandike R, Kramer K, Patrick F, Brown NJ, McElroy PD, Rimisho W, Msengwa A, Mnzava A, Nathan R, et al: **Design, implementation and evaluation of a national campaign to deliver 18 million free long-lasting insecticidal nets to uncovered sleeping spaces in Tanzania.** *Malar J* 2013, **12:**85.
33. Sanchez L, Perez D, Cruz G, Castro M, Kouri G, Shkedy Z, Vanlerberghe V, Van der Stuyft P: **Intersectoral coordination, community empowerment and dengue prevention: six years of controlled interventions in Playa Municipality, Havana, Cuba.** *Trop Med Int Health* 2009, **14:**1356-1364.
34. Sanders KC, Rundi C, Jelip J, Rashman Y, Smith Gueye C, Gosling RD: **Eliminating malaria in Malaysia: the role of partnerships between the public and commercial sectors in Sabah.** *Malaria Journal* 2014, **13:**24.
35. Sharp B, van Wyk P, Sikasote JB, Banda P, Kleinschmidt I: **Malaria control by residual insecticide spraying in Chingola and Chililabombwe, Copperbelt Province, Zambia.** *Trop Med Int Health* 2002, **7:**732-736.
36. Ulibarri G, Betanzos A, Betanzos M, Rojas JJ: **Preliminary results on the control of Aedes spp. in a remote Guatemalan community vulnerable to dengue, chikungunya and Zika virus: community participation and use of low-cost ecological ovillantas for mosquito control.** *F1000Res* 2016, **5:**598.
37. Xu JW, Li Y, Yang HL, Zhang J, Zhang ZX, Yang YM, Zhou HN, Havumaki J, Li HX, Liu H, et al: **Malaria control along China-Myanmar Border during 2007-2013: an integrated impact evaluation.** *Infect Dis Poverty* 2016, **5:**75.
38. Castro MC, Tsuruta A, Kanamori S, Kannady K, Mkude S: **Community-based environmental management for malaria control: evidence from a small-scale intervention in Dar es Salaam, Tanzania.** *Malar J* 2009, **8:**57.
39. Ichimori K, Crump A: **Pacific collaboration to eliminate lymphatic filariasis.** *Trends Parasitol* 2005, **21:**441-444.
40. Deribew A, Birhanu Z, Sena L, Dejene T, Reda AA, Sudhakar M, Alemseged F, Tessema F, Zeynudin A, Biadgilign S, Deribe K: **The effect of household heads training about the use of treated bed nets on the burden of malaria and anaemia in under-five children: a cluster randomized trial in Ethiopia.** *Malar J* 2012, **11:**8.
41. Kittayapong P, Chansang U, Chansang C, Bhumiratana A: **Community participation and appropriate technologies for dengue vector control at transmission foci in Thailand.** *J Am Mosq Control Assoc* 2006, **22:**538-546.
42. Kittayapong P, Thongyuan S, Olanratmanee P, Aumchareoun W, Koyadun S, Kittayapong R, Butraporn P: **Application of eco-friendly tools and eco-bio-social strategies to control dengue vectors in urban and peri-urban settings in Thailand.** *Pathog Glob Health* 2012, **106:**446-454.
43. Sedlmayr R, Fink G, Miller JM, Earle D, Steketee RW: **Health impact and cost-effectiveness of a private sector bed net distribution: experimental evidence from Zambia.** *Malar J* 2013, **12:**102.
44. Wai KT, Htun PT, Oo T, Myint H, Lin Z, Kroeger A, Sommerfeld J, Petzold M: **Community-centred eco-bio-social approach to control dengue vectors: an intervention study from Myanmar.** *Pathog Glob Health* 2012, **106:**461-468.
45. Bhattacharya SK, Dash AP: **Elimination of Kala-Azar from the Southeast Asia Region.** *Am J Trop Med Hyg* 2017.
46. Kusuma YS, Burman D, Kumari R, Lamkang AS, Babu BV: **Impact of health education based intervention on community's awareness of dengue and its prevention in Delhi, India.** *Glob Health Promot* 2017**:**1757975916686912.
47. Tana S, Umniyati S, Petzold M, Kroeger A, Sommerfeld J: **Building and analyzing an innovative community-centered dengue-ecosystem management intervention in Yogyakarta, Indonesia.** *Pathog Glob Health* 2012, **106:**469-478.

48. Zhang J, Dong JQ, Li JY, Zhang Y, Tian YH, Sun XY, Zhang GY, Li QP, Xu XY, Cai T: **Effectiveness and impact of the cross-border healthcare model as implemented by non-governmental organizations: case study of the malaria control programs by health poverty action on the China-Myanmar border.** *Infect Dis Poverty* 2016, **5:**80.
49. Drameh PS, Richards Jr FO, Cross C, Etya'alé DE, Kassalow JS: **Ten years of NGDO action against river blindness.** *Trends in Parasitology* 2002, **18:**378-380.
