# Supplementary material for: Intersectoral collaboration for the prevention and control of vector borne diseases to support the implementation of a global strategy: A systematic review
Source: PLoS One. 2018 Oct 10;13(10):e0204659. doi: 10.1371/journal.pone.0204659 (PMC6179246; doi:10.1371/journal.pone.0204659)
Supplement: S7 Table — (PDF) [file pone.0204659.s008.pdf]

S7 Table. Factors influencing success of intersectoral collaboration

Influencing factors	Component of factors	Author, year	Number of study
Shared vision	Clear agreement on outcomes	[1-20]	20/47
	Proven benefit for each sector	[8, 18, 19]	
	Complete neutrality	[17]	
Management	Strong leadership	[1, 7, 11, 18, 19, 21-23]	29/47
	Strong management and implementation capacity	[1, 4, 5, 11, 17, 18, 24-29]	
	Established committee/working group	[1, 4, 9, 12, 14, 16, 27, 30-32]	
	Joint planning and design program	[4, 6, 9, 11, 16, 17, 19, 21, 30, 31, 33, 34]	
	Close supervision	[4, 6, 8, 12, 18, 19, 24, 25, 34, 35]	
Relationship	Consistent communication	[1, 4, 5, 8, 9, 12, 15, 17, 19, 26, 28, 30, 31, 33, 36-41]	33/47
	Regular meeting	[1, 4, 7-9, 14, 17, 19, 31, 35]	
	Open and transparent	[17, 18]	
	Consistent commitments	[1, 2, 4, 6, 7, 9, 11, 12, 18, 19, 21, 25, 26, 28, 32, 35, 42, 43]	
	Good relationship of trust and cooperation	[2, 4, 7, 9, 12, 17, 28, 41, 44]	
Approach	Use pre-existing organizations or stakeholders	[1, 2, 4, 5, 9-12, 14, 17, 20, 22, 26, 27, 31, 33, 38, 40, 42, 45]	37/47
	Use participatory approach	[1, 3, 4, 6-10, 12, 14-16, 28, 31, 33, 35, 37, 40, 41, 45, 46]	
	Use socio-cultural approach	[1, 9, 17, 20, 35, 38, 47]	
	Empowered community	[1-3, 6-10, 12, 14-16, 28, 30-32, 34, 35, 37, 38, 45]	
	Engagement school teachers and pupils	[1, 2, 5, 8, 10, 12, 14-16, 20, 22, 24, 38, 44]	
Resources	Legislation/policy/local system & norms	[3, 4, 10, 11, 22, 23, 27, 28, 34, 37, 43, 47]	34/47
	Sufficient local funds	[4, 16, 17, 19, 21, 22, 40, 44, 46]	
	Received technical and financial support from international agencies	[4, 5, 8-11, 13-15, 17, 21, 22, 25, 28, 29, 34-36, 42-45]	

Influencing factors	Component of factors	Author, year	Number of study
	Source of funds	[4, 16, 19, 21, 25, 28, 31, 34, 35, 40-43]	
	Stable political situation	[1, 11, 17, 22, 29, 38, 43]	
Type of organizations	Government vs non-government	[41]	1/47

References

1. Abeyewickreme W, Wickremasinghe AR, Karunatilake K, Sommerfeld J, Axel K: Community mobilization and household level waste management for dengue vector control in Gampaha district of Sri Lanka; an intervention study. Pathog Glob Health 2012, 106:479-487.
2. Arunachalam N, Tyagi BK, Samuel M, Krishnamoorthi R, Manavalan R, Tewari SC, Ashokkumar V, Kroeger A, Sommerfeld J, Petzold M: Community-based control of Aedes aegypti by adoption of eco-health methods in Chennai City, India. Pathog Glob Health 2012, 106:488-496.
3. Deribew A, Birhanu Z, Sena L, Dejene T, Reda AA, Sudhakar M, Alemseged F, Tessema F, Zeynudin A, Biadgilign S, Deribe K: The effect of household heads training about the use of treated bed nets on the burden of malaria and anaemia in under-five children: a cluster randomized trial in Ethiopia. Malar J 2012, 11:8.
4. Drameh PS, Richards Jr FO, Cross C, Etya'alé DE, Kassalow JS: Ten years of NGDO action against river blindness. Trends in Parasitology 2002, 18:378-380.
5. Gibbons RV, Nisalak A, Yoon IK, Tannitisupawong D, Rungsimunpaiboon K, Vaughn DW, Endy TP, Innis BL, Burke DS, Mammen MP, Jr., et al: A model international partnership for community-based research on vaccine-preventable diseases: the Kamphaeng Phet-AFRIMS Virology Research Unit (KAVRU). Vaccine 2013, 31:4487-4500.
6. Herdiana H, Fuad A, Asih PB, Zubaedah S, Arisanti RR, Syafruddin D, Kusnanto H, Sumiwi ME, Yuniarti T, Imran A, et al: Progress towards malaria elimination in Sabang Municipality, Aceh, Indonesia. Malar J 2013, 12:42.
7. Ichimori K, Crump A: Pacific collaboration to eliminate lymphatic filariasis. Trends Parasitol 2005, 21:441-444.
8. Kaatano GM, Siza JE, Mwanga JR, Min DY, Yong TS, Chai JY, Ko Y, Chang SY, Kullaya CM, Rim HJ, et al: Integrated Schistosomiasis and Soil-Transmitted Helminthiasis Control over Five Years on Kome Island, Tanzania. Korean J Parasitol 2015, 53:535-543.
9. Kittayapong P, Thongyuan S, Olanratmanee P, Aumchareoun W, Koyadun S, Kittayapong R, Butraporn P: Application of eco-friendly tools and eco-bio-social strategies to control dengue vectors in urban and peri-urban settings in Thailand. Pathog Glob Health 2012, 106:446-454.

10. Kittayapong P, Yoksan S, Chansang U, Chansang C, Bhumiratana A: Suppression of dengue transmission by application of integrated vector control strategies at sero-positive GIS-based foci. Am J Trop Med Hyg 2008, 78:70-76.
11. Krisher LK, Krisher J, Ambuludi M, Arichabala A, Beltran-Ayala E, Navarrete P, Ordonez T, Polhemus ME, Quintana F, Rochford R, et al: Successful malaria elimination in the Ecuador-Peru border region: epidemiology and lessons learned. Malar J 2016, 15:573.
12. Magnussen P, Ndawi B, Sheshe AK, Byskov J, Mbwana K, Christensen NØ: The impact of a school health programme on the prevalence and morbidity of urinary schistosomiasis in Mwera Division, Pangani District, Tanzania. Transactions of the Royal Society of Tropical Medicine and Hygiene 2001, 95:58-64.
13. Martins JS, Zwi AB, Kelly PM: Did the first Global Fund grant (2003-2006) contribute to malaria control and health system strengthening in Timor-Leste? Malar J 2012, 11:237.
14. Tana S, Umniyati S, Petzold M, Kroeger A, Sommerfeld J: Building and analyzing an innovative community-centered dengue-ecosystem management intervention in Yogyakarta, Indonesia. Pathog Glob Health 2012, 106:469-478.
15. Ulibarri G, Betanzos A, Betanzos M, Rojas JJ: Preliminary results on the control of Aedes spp. in a remote Guatemalan community vulnerable to dengue, chikungunya and Zika virus: community participation and use of low-cost ecological ovillantas for mosquito control. F1000Res 2016, 5:598.
16. Wangroongsarb Y: Dengue Control through Schoolchildren in Thailand. Dengue Bulletin 1997, 21:52-62.
17. Zhang J, Dong JQ, Li JY, Zhang Y, Tian YH, Sun XY, Zhang GY, Li QP, Xu XY, Cai T: Effectiveness and impact of the cross-border healthcare model as implemented by non-governmental organizations: case study of the malaria control programs by health poverty action on the China-Myanmar border. Infect Dis Poverty 2016, 5:80.
18. Peters DH, Phillips T: Mectizan Donation Program: evaluation of a public-private partnership. Trop Med Int Health 2004, 9:A4-15.
19. Sanders KC, Rundi C, Jelip J, Rashman Y, Smith Gueye C, Gosling RD: Eliminating malaria in Malaysia: the role of partnerships between the public and commercial sectors in Sabah. Malaria Journal 2014, 13:24.
20. Yuan LP, Manderson L, Ren MY, Li GP, Yu DB, Fang JC: School-based interventions to enhance knowledge and improve case management of schistosomiasis: a case study from Hunan, China. Acta Trop 2005, 96:248-254.
21. Renggli S, Mandike R, Kramer K, Patrick F, Brown NJ, McElroy PD, Rimisho W, Msengwa A, Mnzava A, Nathan R, et al: Design, implementation and evaluation of a national campaign to deliver 18 million free long-lasting insecticidal nets to uncovered sleeping spaces in Tanzania. Malar J 2013, 12:85.
22. van den Berg H, Velayudhan R, Ebol A, Catbagan BH, Jr., Turingan R, Tuso M, Hii J: Operational efficiency and sustainability of vector control of malaria and dengue: descriptive case studies from the Philippines. Malar J 2012, 11:269.
23. Bhattacharya SK, Dash AP: Elimination of Kala-Azar from the Southeast Asia Region. Am J Trop Med Hyg 2017.

24. Afenyadu GY, Agyepong IA, Barnish G, Adjei S: Improving access to early treatment of malaria: a trial with primary school teachers as care providers. Trop Med Int Health 2005, 10:1065-1072.
25. Argaw MD, Woldegiorgis AG, Abate DT, Abebe ME: Improved malaria case management in formal private sector through public private partnership in Ethiopia: retrospective descriptive study. Malar J 2016, 15:352.
26. Ho LL, Tsai YH, Lee WP, Liao ST, Wu LG, Wu YC: Taiwan's Travel and Border Health Measures in Response to Zika. Health Secur 2017, 15:185-191.
27. Kong XL, Liu X, Tu H, Xu Y, Niu JB, Wang YB, Zhao CL, Kou JX, Feng J: Malaria control and prevention towards elimination: data from an eleven-year surveillance in Shandong Province, China. Malaria Journal 2017, 16.
28. Mutero CM, Mbogo C, Mwangangi J, Imbahale S, Kibe L, Orindi B, Girma M, Njui A, Lwande W, Affognon H, et al: An Assessment of Participatory Integrated Vector Management for Malaria Control in Kenya. Environ Health Perspect 2015, 123:1145-1151.
29. Xu JW, Li Y, Yang HL, Zhang J, Zhang ZX, Yang YM, Zhou HN, Havumaki J, Li HX, Liu H, et al: Malaria control along China-Myanmar Border during 2007-2013: an integrated impact evaluation. Infect Dis Poverty 2016, 5:75.
30. Sanchez L, Perez D, Cruz G, Castro M, Kouri G, Shkedy Z, Vanlerberghe V, Van der Stuyft P: Intersectoral coordination, community empowerment and dengue prevention: six years of controlled interventions in Playa Municipality, Havana, Cuba. Trop Med Int Health 2009, 14:1356-1364.
31. Sanchez L, Perez D, Perez T, Sosa T, Cruz G, Kouri G, Boelaert M, Van der Stuyft P: Intersectoral coordination in Aedes aegypti control. A pilot project in Havana City, Cuba. Trop Med Int Health 2005, 10:82-91.
32. Murhandarwati EE, Fuad A, Sulistyawati, Wijayanti MA, Bia MB, Widartono BS, Kuswantoro, Lobo NF, Supargiyono, Hawley WA: Change of strategy is required for malaria elimination: a case study in Purworejo District, Central Java Province, Indonesia. Malar J 2015, 14:318.
33. De Urioste-Stone SM, Pennington PM, Pellecer E, Aguilar TM, Samayoa G, Perdomo HD, Enriquez H, Juarez JG: Development of a community-based intervention for the control of Chagas disease based on peridomestic animal management: an eco-bio-social perspective. Trans R Soc Trop Med Hyg 2015, 109:159-167.
34. Castro MC, Tsuruta A, Kanamori S, Kannady K, Mkude S: Community-based environmental management for malaria control: evidence from a small-scale intervention in Dar es Salaam, Tanzania. Malar J 2009, 8:57.
35. Johns B, Yihdego YY, Kolyada L, Dengela D, Chibsa S, Dissanayake G, George K, Taffese HS, Lucas B: Indoor Residual Spraying Delivery Models to Prevent Malaria: Comparison of Community- and District-Based Approaches in Ethiopia. Glob Health Sci Pract 2016, 4:529-541.
36. Aumentado C, Cerro BR, Olobia L, Suy LL, Reyes A, Kusumawathie PH, Sagrado M, Hall JL, Abeyasinghe R, Foxwell AR, Vestergaard LS: The prevention and control of dengue after Typhoon Haiyan. Western Pac Surveill Response J 2015, 6 Suppl 1:60-65.

37. Chanda E, Masaninga F, Coleman M, Sikaala C, Katebe C, Macdonald M, Baboo KS, Govere J, Manga L: Integrated vector management: the Zambian experience. Malar J 2008, 7:164.
38. Ghosh SK, Patil RR, Tiwari S, Dash AP: A community-based health education programme for bio-environmental control of malaria through folk theatre (Kalajatha) in rural India. Malar J 2006, 5:123.
39. Oyediran AB, Ddumba EM, Ochola SA, Lucas AO, Koporc K, Dowdle WR: A public-private partnership for malaria control: lessons from the Malarone Donation Programme. Bull World Health Organ 2002, 80:817-821.
40. Sharp B, van Wyk P, Sikasote JB, Banda P, Kleinschmidt I: Malaria control by residual insecticide spraying in Chingola and Chililabombwe, Copperbelt Province, Zambia. Trop Med Int Health 2002, 7:732-736.
41. Owusu NO, Baffour-Awuah B, Johnson FA, Mohan J, Madise NJ: Examining intersectoral integration for malaria control programmes in an urban and a rural district in Ghana: a multinomial multilevel analysis. Int J Integr Care 2013, 13:e029.
42. Sedlmayr R, Fink G, Miller JM, Earle D, Steketee RW: Health impact and cost-effectiveness of a private sector bed net distribution: experimental evidence from Zambia. Malar J 2013, 12:102.
43. Njau RJ, de Savigny D, Gilson L, Mwageni E, Mosha FW: Implementation of an insecticide-treated net subsidy scheme under a public-private partnership for malaria control in Tanzania--challenges in implementation. Malar J 2009, 8:201.
44. Okabayashi H, Thongthien P, Singhasvanon P, Waikagul J, Looareesuwan S, Jimba M, Kano S, Kojima S, Takeuchi T, Kobayashi J, Tateno S: Keys to success for a school-based malaria control program in primary schools in Thailand. Parasitology International 2006, 55:121-126.
45. Wai KT, Htun PT, Oo T, Myint H, Lin Z, Kroeger A, Sommerfeld J, Petzold M: Community-centred eco-bio-social approach to control dengue vectors: an intervention study from Myanmar. Pathog Glob Health 2012, 106:461-468.
46. Kittayapong P, Chansang U, Chansang C, Bhumiratana A: Community participation and appropriate technologies for dengue vector control at transmission foci in Thailand. J Am Mosq Control Assoc 2006, 22:538-546.
47. Kusuma YS, Burman D, Kumari R, Lamkang AS, Babu BV: Impact of health education based intervention on community's awareness of dengue and its prevention in Delhi, India. Glob Health Promot 2017:1757975916686912.
