# Supplementary material for: Intersectoral collaboration for the prevention and control of vector borne diseases to support the implementation of a global strategy: A systematic review
Source: PLoS One. 2018 Oct 10;13(10):e0204659. doi: 10.1371/journal.pone.0204659 (PMC6179246; doi:10.1371/journal.pone.0204659)
Supplement: S8 Table — (PDF) [file pone.0204659.s009.pdf]

S8 Table. Barrier to success of intersectoral collaboration

Barrier identified	Author, year	Number of study
Political differences, Political will	[1, 2, 3, 4, 5, 6, 7, 8, 9, 10, 11, 12]	12/21
Financial constraints	[3, 7, 10, 11, 13, 14, 15,]	7/21
Organisational rigidities	[3]	1/21
Contested planning priorities	[10]	1/21
Different organisational histories	[2]	1/21
Different geographic areas	[6, 16]	2/21
Organisational cultures	[3]	1/21
Professional attitudes and behaviours	[10, 17]	2/21
The difficulties of shared decision making and power	[18]	1/21
Inaccessible area	[5, 15]	2/21
Poor leadership	[3, 10]	2/21
Do not see tangible benefits	[12, 15, 19, 18]	4/21
Weak monitoring and evaluation	[3, 10, 20	3/21
Insufficient and irregular supplies	[11, 12, 15, 19, 20]	5/21
Poor communication and coordination	[3, 9, 10, 12, 18, 19, 20, 21]	9/21
Lack of local commitment	[3, 10, 11, 12, 20, 21]	6/21

References

1. Kittayapong P, Yoksan S, Chansang U, Chansang C, Bhumiratana A: Suppression of dengue transmission by application of integrated vector control strategies at sero-positive GIS-based foci. Am J Trop Med Hyg 2008, 78:70-76.
2. Krisher LK, Krisher J, Ambuludi M, Arichabala A, Beltran-Ayala E, Navarrete P, Ordonez T, Polhemus ME, Quintana F, Rochford R, et al: Successful malaria elimination in the Ecuador-Peru border region: epidemiology and lessons learned. Malar J 2016, 15:573.
3. Martins JS, Zwi AB, Kelly PM: Did the first Global Fund grant (2003-2006) contribute to malaria control and health system strengthening in Timor-Leste? Malar J 2012, 11:237.
4. Zhang J, Dong JQ, Li JY, Zhang Y, Tian YH, Sun XY, Zhang GY, Li QP, Xu XY, Cai T: Effectiveness and impact of the cross-border healthcare model as implemented by non-governmental organizations: case study of the malaria control programs by health poverty action on the China-Myanmar border. Infect Dis Poverty 2016, 5:80.
5. Sanders KC, Rundi C, Jelip J, Rashman Y, Smith Gueye C, Gosling RD: Eliminating malaria in Malaysia: the role of partnerships between the public and commercial sectors in Sabah. Malaria Journal 2014, 13:24.
6. van den Berg H, Velayudhan R, Ebol A, Catbagan BH, Jr., Turingan R, Tuso M, Hii J: Operational efficiency and sustainability of vector control of malaria and dengue: descriptive case studies from the Philippines. Malar J 2012, 11:269.

7. Kong XL, Liu X, Tu H, Xu Y, Niu JB, Wang YB, Zhao CL, Kou JX, Feng J: Malaria control and prevention towards elimination: data from an eleven-year surveillance in Shandong Province, China. Malaria Journal 2017, 16.
8. Xu JW, Li Y, Yang HL, Zhang J, Zhang ZX, Yang YM, Zhou HN, Havumaki J, Li HX, Liu H, et al: Malaria control along China-Myanmar Border during 2007-2013: an integrated impact evaluation. Infect Dis Poverty 2016, 5:75.
9. Sanchez L, Perez D, Perez T, Sosa T, Cruz G, Kouri G, Boelaert M, Van der Stuyft P: Intersectoral coordination in Aedes aegypti control. A pilot project in Havana City, Cuba. Trop Med Int Health 2005, 10:82-91.
10. Murhandarwati EE, Fuad A, Sulistyawati, Wijayanti MA, Bia MB, Widartono BS, Kuswantoro, Lobo NF, Supargiyono, Hawley WA: Change of strategy is required for malaria elimination: a case study in Purworejo District, Central Java Province, Indonesia. Malar J 2015, 14:318.
11. Castro MC, Tsuruta A, Kanamori S, Kannady K, Mkude S: Community-based environmental management for malaria control: evidence from a small-scale intervention in Dar es Salaam, Tanzania. Malar J 2009, 8:57.
12. Kusuma YS, Burman D, Kumari R, Lamkang AS, Babu BV: Impact of health education based intervention on community's awareness of dengue and its prevention in Delhi, India. Glob Health Promot 2017:1757975916686912.
13. Gibbons RV, Nisalak A, Yoon IK, Tannitisupawong D, Rungsimunpaiboon K, Vaughn DW, Endy TP, Innis BL, Burke DS, Mammen MP, Jr., et al: A model international partnership for community-based research on vaccine-preventable diseases: the Kamphaeng Phet-AFRIMS Virology Research Unit (KAVRU). Vaccine 2013, 31:4487-4500.
14. Magnussen P, Ndawi B, Sheshe AK, Byskov J, Mbwana K, Christensen NØ: The impact of a school health programme on the prevalence and morbidity of urinary schistosomiasis in Mwera Division, Pangani District, Tanzania. Transactions of the Royal Society of Tropical Medicine and Hygiene 2001, 95:58-64.16.
15. Afenyadu GY, Agyepong IA, Barnish G, Adjei S: Improving access to early treatment of malaria: a trial with primary school teachers as care providers. Trop Med Int Health 2005, 10:1065-1072.
16. Herdiana H, Fuad A, Asih PB, Zubaedah S, Arisanti RR, Syafruddin D, Kusnanto H, Sumiwi ME, Yuniarti T, Imran A, et al: Progress towards malaria elimination in Sabang Municipality, Aceh, Indonesia. Malar J 2013, 12:42.
17. Abeyewickreme W, Wickremasinghe AR, Karunatilake K, Sommerfeld J, Axel K: Community mobilization and household level waste management for dengue vector control in Gampaha district of Sri Lanka; an intervention study. Pathog Glob Health 2012, 106:479-487.
18. Oyediran AB, Ddumba EM, Ochola SA, Lucas AO, Koporc K, Dowdle WR: A public-private partnership for malaria control: lessons from the Malarone Donation Programme. Bull World Health Organ 2002, 80:817-821.
19. De Urioste-Stone SM, Pennington PM, Pellecer E, Aguilar TM, Samayoa G, Perdomo HD, Enriquez H, Juarez JG: Development of a community-based intervention for the control of Chagas disease based on peridomestic animal management: an eco-bio-social perspective. Trans R Soc Trop Med Hyg 2015, 109:159-167.
20. Wangroongsarb Y: Dengue Control through Schoolchildren in Thailand. Dengue Bulletin 1997, 21:52-62.

21. Mutero CM, Mbogo C, Mwangangi J, Imbahale S, Kibe L, Orindi B, Girma M, Njui A, Lwande W, Affognon H, et al: An Assessment of Participatory Integrated Vector Management for Malaria Control in Kenya. Environ Health Perspect 2015, 123:1145-115
